# Analectic

**Published:** 1831

**Authors:** 


### MISCELLANEOUS INTELLIGENCE.

### ANALECTIC, ANALYTICAL, AND ORIGINAL.

### I. ANALECTIC.

1. *The Nature and Cure of the Indian Cholera.*—It behooves every medical practitioner to watch with eagle eye the progress of this dreadful malady, and to treasure up in his mind every incident in its history which may aid in forming philosophical views with regard to its treatment. It will not therefore be with indifference that the reader will peruse the following remarks, which we derive from a late number of that able London periodical, "the Englishman's Magazine."

The Indian or Spasmodic Cholera, is a disease quite distinct from the Bilious Cholera which is common in this country and in Great Britain in hot seasons, and the author commences by pointing out the distinctive characters of these two diseases.

The generic appellative, *cholera*,* is radically derived from a Greek word signifying bile. The title was appropriated in consequence of one of the most prominent symptoms being either a redundant flow of bile into the intestines, accompanied with evacuations of a *bilious fluid*, characterizing *Bilious Cholera;* or a total absence of bile in the intestines, with evacuations of a *watery fluid*, characterizing *Spasmodic Cholera.* These symptoms are so completely opposed to each other in the quality of the fluid discharged, that attending to them alone, will, in general, preclude the possibility of confounding the two species.

In this country, the more severe forms of Bilious Cholera usually occur in the latter end of summer, or the beginning of autumn. At these periods, the extreme heat of the sun stimulates the liver to an increased secretion of bile, which, flowing in excess through the biliary canals, accumulates within the intestines. In the condition of health, a certain quantity of bile is a necessary adjuvant to the perfecting of the digestive process; but, like other natural secretions, when above or below the salutary measure, it becomes the cause or the indication of diseased action.

---

* Cholera *Morbus*, the vulgar name, is an absurd pleonasm.

The opening symptoms of Bilious Cholera, are simply the efforts of nature to expel the superfluous bile from the system. The patient complains of nausea and universal languor, to which, retchings and evacuations of a bilious fluid quickly succeed. There is also thirst, restlessness, and pain. In many cases, occasional cramps are experienced in the limbs, resulting doubtless from the irritating effects of the bile upon the internal surface of the intestines. Of spasmodic action, induced in different parts of the body in an analagous manner, there is conclusive evidence. In children, convulsions are often excited by the irritation of worms, or indigestible matter lodged in some part of the alimentary canal.

A knowledge of the nature and seat of a disease will lead to a judicious plan of medical treatment. The course to be adopted here is very obvious. There are two objects to be attained. One, to dilute and remove the irritating fluid from the bowels; the other, to allay the thirst, pain, and spasms. The first indication is followed in the free exhibition of mucilaginous liquids, and the use of an occasional purgative; to compass the second, anodyne medicines and refreshing drinks should be administered. When properly treated in the beginning, it is very seldom indeed, under ordinary circumstances, that cases of Bilious Cholera prove fatal. The disease is acute for the time it endures, but generally it is subdued in three or four days, and the patient recovers with nearly the same rapidity that he fell sick. There are periods upon record, however, in which the malady assumed a more dangerous tendency, in consequence, probably, of a peculiar constitution of the atmosphere with regard to heat, moisture, &c., or properties unknown. In 1669, Sydenham describes the Bilious Cholera as unusually prevalent and severe, carrying off many victims in the space of twenty-four hours.

*Spasmodic Cholera*, is strongly distinguished from the preceding species by the intractable nature of the malady itself, and by the greater intensity of some symptoms common to both, as well as by the features, which confer upon it a distinct specific existence. Spasmodic Cholera is practically unknown in England, but in India it is indigenous. The attack is usually most insidious and sudden. Persons who may have felt perfectly well during the day, in the course of the night or early in the morning are seized with a feeling of uneasiness, which is at first rather a general sensation than referable to any part. To this succeed, at irregular intervals of time, a sensation of heat in the region of the stomach, nausea, constant evacuations from the stomach and bowels of a fluid bearing a striking similitude to water in which rice had been boiled, cramps of the muscles of the fingers and toes, which gradually ascend along the limbs to the trunk. Finally, the muscles of the chest and belly are included in the circle of spasmodic movement, the more violent symptoms continue to persecute the patient, until his strength is unable longer to sustain them. In the last stage of the disease, he is emancipated from the vomiting and spasms, through the complete exhaustion of physical power. With this change, however, he expresses himself greatly relieved, and he may yet survive for a considerable period, his mind remaining unimpaired amid the wreck of the corporeal functions.

A physician conversant with cases of Spasmodic Cholera, will often be competent to recognize an approaching attack in the expression of the patient's face, before that he is himself conscious of the least alteration in his appearance or sensations. His features seem sharper than natural, and there is an air of repressed anxiety in his countenance. If his attention be called to the fact, he will then perhaps say that he does not feel altogether as easy as in general, but that he cannot account for the impression. The changes which the pulse and skin undergo, in the more obvious stages of the malady, are very remarkable, and will not fail to strike the attention of the most superficial observer. At first, the pulse is rapid, small and weak. When the spasms are established, it becomes, during the paroxysms, imperceptible in the limbs, and for some time before death no pulsation can be detected in these parts. The inability to carry on the circulation in the superficial textures, allows the blood to accumulate and oppress the internal organs. On post-mortem examination, the different viscera, particularly the liver and lungs, are found gorged with thick, dark-colored blood, showing that the eliminating processes of respiration and secretion had been very imperfectly performed. The surface of the body, at the commencement of the disease, is pale, chilled, and clammy. In the advanced stages, it is quite cadaverous.

The first case of Spasmodic Cholera that challenged our professional skill, occurred at Calcutta. The symptoms were sufficiently marked to make a lasting impression upon the mind of a practitioner hitherto practically unacquainted with an enemy so obstinate and so accustomed to triumph.

The patient, Mr. A——, an European, only a few months transplanted from England, was a middle-aged man, of spare, but muscular proportions. The evening previous to the attack, he had been abroad enjoying the society of some friends, and, contrary to his general habits, he had indulged in the rather free use of spirituous liquors. The party separated about one o'clock. Mr. A——, the distance being short, travelled home in a palankeen. Upon reaching his residence, he did not retire directly to bed, but took a seat in the verandah "to cool himself." In this state of exposure to the night air, he fell asleep. He slept soundly until awakened, an hour afterwards, by his servant, who reminded him of the propriety of going to bed. Either from the disease not being perceptibly manifested, or from impaired sensibility, Mr. A—— was not then conscious of any morbid affection. After slumbering in bed, however, for a couple of hours, he awoke suddenly with a start, complaining of mental anxiety, and a feeling of uneasiness at the region of the stomach, which he attributed to unpleasant dreams, and to the effects of wine. But the anxiety increased, and the uneasiness changed into a feeling of burning heat. In the lapse of four hours, evacuations of the portentous fluid, like rice water, followed, from the stomach and bowels, to which were soon superadded distressing cramps, affecting the muscles of the toes. The character of the affection was now evident to the patient himself, though almost a stranger to the climate and its diseases. To afford medical assistance, we were immediately summoned. The summons was quickly obeyed, but, in the interim, the advance of the disease had surpassed our rapidity.

On arrival, we found that the patient had been unable longer to endure confinement in bed. The cramps had extended upwards to the calfs of the legs; they were also simultaneously experienced in both arms. So violent were the spasms, that he had rolled in torture upon the floor. At the time we entered, he was gathered into a corner of the apartment, and he presented an appalling spectacle of internal agony. His person, sparingly concealed in a night garment, bore, in its spasmodic contraction, a resemblance to the letter S. Assisted by a couple of servants, and by pressing his bent extremities against the angular walls, he labored in the extreme exertion of voluntary force to subdue the involuntary action of the rebellious muscles. The expression of his face at that moment, lives distinct in our recollection; and even there, though seen through the mist of receding years, it is painful to dwell upon.

To convey to the reader a faint idea of the death-struggle then maintained, we should liken Mr. A—— to a traveller, who, falling unwarily upon the tiger's lair, rallies every nerve to secure a temporary respite. His inevitable fate is briefly procrastinated by the convulsive grasp which holds the throat of the savage in momentary subjection. Such was the danger—such the desperation stamped upon the countenance of our patient. His features were sharp and hollow. His teeth clenched in breathless agony. The blood had retreated from his cheeks and lips. His limbs were doubled resistlessly by the remorseless spasms.

In a few minutes, a remission brought partial relief. In occasional snatches of expression, he gave us to understand that he was now able to answer the interrogatories we might think proper to propose, with respect to the inward symptoms. These symptoms were the usual concomitants of Spasmodic Cholera in the intermediate stage of its violence. His stomach felt as if it contained a furnace. The thirst was unslakeable. And so complete was the feeling of exhaustion during the interval of mitigated suffering, that he felt unconscious of possessing the slightest control over the motions of any part of his physical structure.

Medical men have been collectively accused, by the indiscriminating multitude, of defective sympathy towards the sons and daughters of bodily affliction. Habituated, it is said, in their daily avocations, to the appeal of the unfortunate, they are at last led to conceive the heart-pang of the patient to be as unsubstantial as the words in which it is expressed. The charge is untrue. In no class of educated people will there be found a greater proportion of " hands open as day to melting charity," than might be discovered among the members of the medical profession, were their kind acts performed in the market-place, instead of the gloomy recesses of morbid destitution. Men in the general walks of life may annually exhibit their measured benevolence in public places, and shed the unfrequent tear of commiseration in their hasty transit through a hospital; but it is the province of the practitioner to do something more than this. It is his duty to linger long with the distressed, to bind in solitude their bleeding wounds, and when hope has departed, never to return, to wear her cheering portraiture, that the abrupt and rugged path leading to dissolution may not too hastily reveal the extremity of danger.

To our patient, laboring under a violent and advanced attack of Spasmodic Cholera, no solid expectation of recovery could be extended. Every means, however, sanctioned by recorded experience, was tried to compass a favorable change. Respecting the final issue, he was himself little if at all solicitious. The immediate suffering, particularly from the spasms, absorbed both sense and soul. He prayed imploringly to be relieved, either by energetic treatment or by death, from the intolerable cramps that threatened to tear him into pieces.

Brief was the interval between supplication and repose. The resources of nature were exhausted in detail. The spasms which had eventually ascended to the body, finally yielded to debility, that proved alike prostrating to the actions natural and diseased. The vomiting ceased to harass, the pulse was no longer susceptible in the limbs; even the motion of the heart—that citadel of life, was feeble and indistinct. The surface of the body felt cold and clammy like a corpse, presenting on the hands and feet a corrugated and macerated appearance, as if it had been steeped in water for some days.—The breath grew chill. The eye was glazed. In this state, notwithstanding, he lived several hours, and then expired without a struggle.

The case of Mr. A—— is a common specimen of the progress of the disease to a fatal termination. It included thirteen hours from the first feeling of uneasiness, until he breathed his last. Cases, however, are continually occurring, in which the duration of the malady extends to twenty-four hours. Some beyond that. On the other hand, many patients are carried off with singular rapidity. In the history of the Epidemic, instances are numerous of soldiers falling in the line of march and dying instantly, as if seared by lightning, without having uttered a previous complaint. Mechanics have perished with their working implements in their hands; the Brahmin, also, at his beads, and the Ryot at his plough.

Before mentioning the principles hitherto most successfully pursued in the medical management of Spasmodic Cholera, we shall briefly advert to some of the opinions entertained respecting the causes, remote and immediate, of that terrible distemper. That the reader may comprehend our technicalities of causation, the application of the terms enjoined shall be explained.

The causes of disease admit of two chief divisions. The *remote*, and the *immediate* or *proximate*. It may be permitted for our purpose to illustrate these in the following manner:—A man receives a blow from a stone; the part stricken is bruised; inflammation of the part succeeds. Here the propelled stone is the remote cause of the mischief; the bruise is the proximate cause; and the consequent inflammation forms an array of symptoms, or what is commonly called the disease. In strictness of language, however, we should not call the inflammation, or third stage, the disease itself; it is merely symptomatic of the previous organic change comprised in the second stage. The proximate cause, therefore, is the real disease, the parent of all that follows. Applying this mode of investigation to the English or Bilious Cholera, it will appear that the heat of the sun is the remote cause of the affection; for, during the maximum of its annual range, the heat stimulates the liver to increased action, and its function becomes accelerated. This functional derangement is the proximate cause; and the augmented flow of bile is merely one of the primary symptoms which, in its turn, gives rise to the secondary train, nausea, vomiting, &c.

As in the two examples just given, every malady will have for its proximate cause either an organic change, or a functional derangement of some part of the body. But in many cases we are not aware of the precise nature of that change, or the exact seat of that derangement. It is also often impossible, from the minuteness of the field of research, and the imperfection of our senses, to ascertain, by observation or dissection, whether the proximate cause of certain maladies should be referred to alteration of structure, or to supernatural action alone. To this order belong a numerous family of fevers, which are developed in the system spontaneously, or through the influence of a deleterious miasm. The difficulty to which we have alluded, accounts for the various controversies maintained of late years, among medical writers, regarding the primary seat of fever. Some attempted to prove portions of the nervous, others, of the vascular system, to be the first *link* in the chain of febrile transition. A third party, observing that the brain, in persons who died of fever, frequently presented the aspect of recent inflammation, hastily referred the proximate cause to inflammation of the brain. A fourth, for similar reasons, found it in the lining membrane of the stomach. The more enlightened view, however, of the pathology of fever, demonstrates that the inflammatory appearances observable after death, are the *effects*, and not the cause, of the disease. An opinion, consequently, has been with reason entertained, that the nervous system, not in part, but as a whole, is the radical seat of the morbid phenomena.

The recorded history of Spasmodic Cholera does not reveal to us the reason of the disease changing its character in 1817. Why it should have assumed, at that period, an aspect of extraordinary malignity, has not as yet been satisfactorily explained. The remote and proximate causes of the epidemic are still open to investigation, though several authors have attempted to point them out. Mr. Annesley* seems to think that the remote cause was a peculiar state of the atmosphere, with regard to electricity; that the air was negatively electrical, and that this induced a great diminution of the nervous fluid in the human body, which is in his opinion the proximate cause of Epidemic Cholera.

* Sketches of the most prevalent diseases in India. London: 1829.

Thirteen years have elapsed since Cholera commenced its ravages, and a single experiment cannot be adduced to countenance the existence of the peculiar non-electoral state of the atmosphere. Holding, therefore, his opinion to be perfectly gratuitous, we shall leave Mr. Annesley to the enjoyment of the more solid honors which have been awarded him in the practical department.

Several theoretical writers agree with the practical, in supposing that immediate and increasing diminution of the nervous energy, is the proximate cause of the malady. Dr. Good* does not express his sentiments fully and explicitly upon the subject, but from the little that he says, and as he leaves the opinion uncontroverted, it may be presumed that he was inclined to the same conclusion. Of the remote cause, Dr. Good offers no solution.

To dissent from highly respectable authorities, may be considered in us an act of temerity, both on account of the difficulties appertaining to the question itself, as well as the numerical danger incurred in opposing, to the opinion of the many, that of an individual. Appealing, however, to facts admitted by all, and elucidating these through the light of a clear analogy, we hope to be able to show that, during the first stage of Spasmodic Cholera, the nervous system is not in a state approaching to exhaustion; but, on the contrary, that *it is locally in excess, and to the influence of this excess upon certain parts of the body should the phenomena be ascribed, which externally mark the disease.*

The prevalent opinion, that debility is the immediate cause, probably originated in the medical observers drawing their conclusions erroneously from the effects of the symptoms of Cholera, as had previously been done by others with regard to fever. But it is evident, that in quest of the fountain we should not follow the river to the sea. In whatever stage of the disease the investigation be commenced, we must take the symptoms individually, and ascertain their relations and priority of origin. Guided in this way by the landmarks of observation, we shall finally arrive at a knowledge of the first sensible indication of the morbid action which had been excited within the body, and the next step will be to determine, with the assistance of experience and analogy, the nature of the insensible or hidden derangement, which constitute the proximate cause.

If general nervous debility be the cause of the symptoms, we would ask how it comes to pass that the mind of patients, laboring under Spasmodic Cholera, remains perfectly clear and collected to the last, after the pulse has ceased to vibrate in the limbs, and when death has crossed the threshold of existence? In typhous fever it is otherwise. Here the decline of the cerebral function is evinced in low muttering delirium; in the loss or perversion of the external senses. The ear is mocked by the imaginary fall of waters; the eye deceived by the unsubstantial creations of the brain. The medical treatment, also, found most efficacious in opposing the onset and progress of Cholera, demonstrates that general nervous debility cannot be the cause of the

---

* Study of Medicine, by John Mason Good, M. D. 1829.

earlier symptoms. When a patient is first seized, a copious bleeding from the arm, and a large dose of laudanum, are the remedies chiefly to be depended upon, and these, if resorted to on the approach of the malady, will be generally successful in checking or moderating its progress.

It may be alleged, "the principle of this curative process is decidedly stimulant; the abstraction of blood, instead of depressing the powers of life, tends to strengthen them, breaking the chain of diseased action, and relieving the heart, already oppressed, of a portion of its circulating load; brandy, laudanum, and other narcotics," it may be continued, "are stimulants in the first instance, their narcotic influence being a secondary effect, and therefore they are often successful in counteracting debility in diseases, of which nervous prostration is, doubtless, the most prominent feature." In reply to these arguments, we can perceive no parallel existing between the treatment suited to a disease of strict nervous debility, such as typhous fever, and that of Spasmodic Cholera. In the cases of the former, where the putrid tendency is developed with the accession, to resort to blood-letting will diminish the chances of recovery to almost nothing. The copious and repeated administration also of narcotic medicines, that prove our sheet-anchor in withstanding the inroads of Cholera, would rapidly extinguish the flickering ray that sheds vitality in typhus.

That local excess of nervous energy is the cause of the symptoms, is supported by the pathology of Cholera. The localities in which this excess is generated, appear primarily to be the nerves connected with the liver, stomach and bowels. The nerves of the limbs, and of the other parts, subjected to spasm, are probably secondarily affected. The effects correspond to the cause. There is uneasiness at the pit of the stomach, and the patient's face wears the anxious and shrunk expression common to severe abdominal distempers. The canal, along which, in health, the bile freely travels to the intestines, is closed by the constriction of cramp. The spasms soon extend to the stomach and bowels, and they, in consequence, are compelled to evacuate their contents. The patient now complains, for the first time, of extreme exhaustion. This is worthy of special remark as corroborative of our views, that debility is an effect, and not the cause, of the earlier symptoms.

The character of the attendant spasms would not lead us to suspect a diminution of energy in the nerves distributed to the muscles affected. They are of the rigid kind. Is inordinate and continued action, then, the result of debility? Does deficiency of stimuli excite muscles of extraordinary contractility? Surely not. That the patient will be reduced to a state of helpless exhaustion, is no reason against entertaining the previous redundance of nervous influence. The concord of the animal functions, voluntary and involuntary, which characterizes health, is broken by the undue accumulation of energy in any one of the textures subservient to them. Nor do the instances in which Cholera proves almost instantaneously fatal, militate against our position. They only show that a stimulus, which in a common degree of activity excited undue action alone, will in a greater measure completely destroy it. Thus electricity, in graduated shocks, may recruit, or derange, or destroy the body. In the same way, cold, under ordinary circumstances, is a bracing stimulant, but in excess it is dangerous to life. Mr. Scott has recorded a curious case, which, in so far as the nerves of the extremities are concerned, bears hard upon the doctrine of debility. It is that of a man who had been subject to paralysis and total numbness of his limbs. In addition, he had the misfortune to be seized with Spasmodic Cholera, when, to the surprise of his attendants, his limbs became the seat of spasms, and also exquisitely sensible.

The possibility of effecting a cure in Spasmodic Cholera, greatly depends upon the time in which the patient is submitted to medical management. Should the disease be allowed to completely develope itself before advice is obtained, it will frequently baffle the exertions of the most skilful practitioner, and prove rapidly fatal. But if the physician be consulted when the symptoms are moderate, when uneasiness and anxiety are chiefly complained of, after the use of the customary remedies, strong hopes of recovery may be indulged. Blood-letting, and a large dose of calomel, should be immediately prescribed. To these should succeed constant frictions of hot flannel to the skin; internal prescriptions of laudanum, brandy, and water, and other sedative anti-spasmodics, to be used at such intervals of time, and in such quantities, as the physician may consider best fitted to the peculiarities and urgency of the case. The signs of returning health will be recognized in the reappearance of bile in the evacuations. The secretions of saliva and of urine, which had been suspended during the severity of the attack, will be again restored. The breath and skin will gradually recover their natural heat; and, in short, every function of the system will return to the salutary standard.

Should the patient delay application for advice until the disease has advanced considerably into the second stage, venesection will generally prove useless or injurious. The cramps are established, and they should be alleviated by friction, and the exhibition of anti-spasmodic medicines. If the third or last stage have set in, a discriminating judgment must also be exercised. As debility has now become our only opponent, the sedative preparations are to be modified accordingly, so as to produce little more than a stimulant effect; for the use, at this period, of laudanum, &c., in quantities suited to the treatment of spasms, would render the catastrophe inevitable.

Although we are unacquainted with the individual remote cause that imparted to Spasmodic Cholera the epidemic and fatal character which it assumed in the town of Jessore during 1817, it is clear that it must have depended upon one of the three following circumstances:—1st. Either some peculiarity in the condition of the atmosphere; 2d. Or some peculiarity in the condition of the human body; 3d. Or upon a peculiar condition of both. As the various writings upon the subject do not rise above speculation, we shall pass over them to inquire in what manner the disease is propagated in the present day; whether it is communicated from one person to another by contagion? Or whether the atmosphere is the sole remote agent in exciting the distemper?

A few years ago Dr. Maclean divided the medical world into contagionists and non-contagionists. Our sentiments are decidedly opposed to those of Dr. Maclean; yet, notwithstanding, we suspect that the attention he received at the hands of the College of Physicians, was not commensurate to his abilities. The public, moreover, through sheer ignorance of the steps leading indirectly to the temple of science, whilst it swallows with avidity the monsters of quackery *practice*, is ever ready to raise an idle clamor against theories—medical theories in particular.

> To doubt the sun's a sea-coal fire,
> Would mightily displease
> Some folk, who think the whey-faced moon
> Is made of recent cheese.

"In the world of wisdom theories abound." Prosecuted by men of abilities, even false theories are often productive of much good. The hasty growth of the structure, lacking bone and muscle, either destroys itself, making men wiser by experience; or it demonstrates how far the theory, which has entered upon the right road, may be received. Dr. Maclean failed to prove the non-contagious nature of Egyptian plague, but the College of Physicians were obliged to admit that some modification, advantageous to the interests of commerce, might be made in the quarantine laws.

The fundamental error which governed Dr. Maclean, was an extravagant love of uniformity. He forgot that Nature, while she carefully preserves the family likeness, frequently leaves details to the "beauty of contrast." His first grand position* was, that fevers truly contagious could not affect the same person more than once in a natural life. Therefore, Egyptian plague and typhous fever were non-contagious. Secondly, that epidemics are not propagated by contagion, but depend upon atmospheric causes. Unmindful that he was himself the maker of these "laws," he called them fixed and unchangeable.

The distinction drawn between epidemic and contagious diseases, was altogether fanciful. The fact is, that contagious diseases may become epidemic; and epidemic diseases, originally dependent upon atmospheric causes, may become contagious. Scattered cases, for instance, the smallpox—a disease, the contagion of which is unquestioned—are constantly occurring in various parts of England; but occasionally it attacks great numbers of children, about the same time and place, or, in other words, it assumes the epidemic form. The contagious fever of measles is obedient to similar laws.

Contagious diseases are communicated from one person to another in two ways, either through the medium of contact, or by close exposure to exhalations emanating from a person infected. To avoid a

---

* Dr. Maclean on Epidemic and Pestilential Diseases, 1817. Ditto on the Evils of Quarantine Laws, 1824.

war of words, these affections may be said to have an *animal origin.* Diseases, on the other hand, that spread, independent of contact or animal exhalations, may be said to have an *atmospheric origin.* The task of arranging these maladies under two distinct heads, in relation to their causes, appears to the uninitiated a work of extreme simplicity. But it is not so; for, as we stated above, they do not always retain the identical character that distinguished their first appearance. Several fevers vary in their cause. The same disease may at one time be referred to the atmospheric, at another to the animal origin.

Egyptian plague furnishes an example of the variable remote cause to which we allude. This disease prevails during the winter half of the year in Lower Egypt. It is highly contagious; but though communicated rapidly from individual to individual, by exposure to pestiferous contact or exhalation, there is sufficient evidence to show, that persons resident there may contract the disease without having undergone any such exposure, and that afterwards they may, by contagion, transmit it to others. Now, a physician who had witnessed a few of the spontaneous cases, if we may so call them, judging from his own limited experience, might ascribe every instance of the disease to an atmospheric origin exclusively; while another, who had only met with instances the result of contagion, might ascribe every case as exclusively, and with equal justice, to an animal origin. This view of the matter may diminish the astonishment with which the public behold the discrepancies of medical evidence.

The admission of a variable remote cause for some contagious diseases, is forced upon us at home in the history of typhous fever. It is commonly propagated by a specific contagion; yet certain external circumstances are sufficient of themselves to generate the disease in the human body. These are, damp houses, ill-ventilated, and crowded with inhabitants; wet winters, scarcity of food, and all the depressing concomitants of poverty. In this way have originated hospital, camp, and jail fevers, which are of a contagious nature, and may be collectively included under the appropriate name of *typhus*.

The digression upon the animal and the atmospheric origin of Egyptian plague and typhous fever, was introduced to prepare the reader for a few observations regarding the origin of Spasmodic Cholera. While the disease was restricted to Hindoostan and its neighborhood, the members of the medical profession in India endeavored to discover the medium through which it was propagated. A large majority, more particularly of the Bengal Presidency, declared the disease to be non-contagious. But in Bombay, the contrary conclusion was ably maintained. In justice to the early advocates of non-contagion, it should be observed that the question, at the period to which we refer, wore a somewhat different aspect from that which it assumed under the impression of later events. The progress of Cholera had then scarcely exceeded the boundaries of Hindoostan, and here the mild and malignant varieties were dangerous. The non-contagionists might, therefore, reasonably ask, "why a disease which began at Jessore, independent of contagion, should not likewise be generated in other localities under the influence of atmospheric causes?" We believe that it was so generated in many instances, and upon the strength of this admission we would reconcile several apparently conflicting statements; the leading features, however, developed in the history of the malady, cannot be satisfactorily explained, in the absence of contagion, on any known accidental condition of health and of the atmosphere. These features are—

1st.—Epidemic Cholera* has travelled as often against, as with the course of the winds. In the very face of a strong S. W. wind which blew in that direction for some months, it passed from Bengal to the Deccan. It has prevailed in every kind of weather common to the climates affected. In the dryest weather, and during the deluge of periodical rains; in storms, and in calms; under the scorching sun of Arabia, and amid the snows of Russia.

Opposed as are these facts to the usual progress of maladies, the extension of which depend solely upon the atmosphere, the character of the succeeding, favors, in a still greater degree, the existence of a contagious power.

2nd.—Epidemic Cholera has in general rigidly followed the great highways of human intercourse. Pursuing the line of navigable waters, and the route of caravans, it entered or traversed the different countries. Through India it extended along the rivers Ganges, Hooghly, Jumna, and Nerbudda. Arabia, Persia, and Syria, were penetrated by the Persian Gulf, the Tigris, and the Euphrates. Moscow received the disease by the route of the Volga. China, other parts of Eastern Asia, and the various islands, were infected over sea, as appears from the Cholera making its earliest ravages in the port towns and maritime districts. Agreeing with the disposition of contagious diseases, the Cholera has been most virulent wherever human beings were numerous and concentrated. In densely-peopled cities; in armies encamped, or upon the march; in localities unfavorable to free ventilation,—as low sheltered grounds, narrow streets, close dirty houses. The slow rate of progression at which the epidemic advanced from place to place in succession, and the temporary halts which it occasionally made, perfectly agree with a contagious origin; but they cannot be reconciled to an atmospheric. It travels, on an average, at a rate varying between ten and eighteen miles a day. But often, in particular instances, much less. Within the Zillah of Nellore it proceeded thirty-two miles in twelve days; in the next twenty-seven days, eighty miles.

Writers who deny the contagious nature of Cholera, rest their belief chiefly upon the circumstance that many persons were attacked without having had previous intercourse with the sick. This objection brings but little weight with it. In Hindoostan such cases may have, at times, arisen from external causes, as at Jessore; but in other countries, where Spasmodic Cholera has never been known until the Indian invasion, we would refer them to contagion, for it is

---

* *Vide* the different Reports compiled by order of the East India Company; and also the publications of individuals upon the subject.

notorious that contagious exhalations may be carried about in merchandise, clothing, &c., their infecting energy remaining for a considerable period unimpaired.

The Russian government was of opinion that the Cholera, during 1829, had entered the province of Orenburg with the caravans trading between Orenburg and Boukhara, the commercial entrepot of Central Asia. The Russians, indeed, have uniformly treated the disease as if contagious. The medical council of Petersburgh issued quarantine orders, under which every patient was to be strictly prohibited from holding close intercourse with persons in health. Even the Emperor Nicholas, who to encourage the inhabitants, visited Moscow during the prevalence of the Cholera, underwent, before his return to Petersburgh, the usual ordeal of purification in quarantine. How far these precautions are productive of benefit, it is difficult to say. The disease was equally mortal in Russia as elsewhere, comparing the number of deaths with the number of the diseased; but it is a remarkable fact, that fewer of the people by far were attacked there than in southern countries. Whether this partial immunity resulted from the influence of climate, and the stronger constitutions of the Russians, or the rigid quarantine, or from a combination of these three circumstances, it is, perhaps, impossible at present to decide. In the island of Bourbon, however, where sanatory regulations were prescribed and enforced, the malady spread less extensively than in the neighboring island of Mauritius, in which these things were neglected. As the character of the inhabitants and of the climate is similar in both islands, this fact is in favor of the utility of quarantine; but the strongest evidence in support of contagion, and the propriety of enforcing quarantine, is, that the appearance of the disease in one country or district, has been generally shown to have soon succeeded to the arrival of persons from another, in which the epidemic had prevailed. In Persia, the gates of Ispahan were closed against the suspected caravan. It consequently passed through Yezd. Shortly afterwards the Cholera destroyed 7000 of the inhabitants of Yezd, while the former city escaped.

Cholera is capricious in the selection of its victims. The infirm and debilitated are its favorite subjects. Yet the best state of health will not ensure exemption. This is not opposed to our view of the proximate cause; debility renders the system more susceptible of morbid impressions,—be they sedative or be they stimulant. The black population suffers in a greater proportion than the white. It is calculated that four millions of the natives of India have been swept away by the scourge since 1817. A share of the mortality, however, may be fairly attributed to partial or total want, in a variety of instances, of medical assistance. In one district, the population of which is about 200,000 souls, the cases of Cholera amounted to 15,945; of these, 1,294 had been without medicine or medical aid, and there is reason to believe that of the number every individual perished. When proper medical means could be supplied at an early period, and their use continued, the result was gratifying to the friends of humanity; and creditable to the profession, if the intractable nature of the malady be taken into account. The Madras army consisted of 83,336 men, European and native. During 1818, and the four succeeding years, there died 5 1-3 per cent of the whole force; or 23 3-4 per cent of those who had been attacked by the epidemic.

The laws of Cholera bear the impress of that presiding Intelligence who has described a circle, beyond which every species of physical evil must cease to mar the harmony of life. Were the disease to continue its ravages in the same place uninterruptedly for a series of years, it would depopulate the world. Few localities have suffered longer than four or six weeks at a time, under the worst form of the distemper, and to this succeed long intervals of safety more or less complete. It does not, moreover, promise to be a plague that will descend a miserable inheritance to many generations. Some countries, formerly afflicted, are even now returning thanks for permanent relief; and in most, the destroyer has relaxed its severity. Wherever it may next direct its course, the principal danger is to be apprehended. May its footsteps be averted from the dwellings of the poor in Great Britain and Ireland!*

2. *Several Cases of Spinal Irritation.* By David Wark, Surgeon, Dunlop.—The subject of spinal irritation, has, of late, attracted considerable attention, but so far as I have had an opportunity of judging of the opinions of practitioners, and from the paucity of writings on the subject, its occurrence would appear to be considered by the greater part of the profession, as but rare, and the violence of its symptoms by no means extreme. During the short term of little more than four years' practice, I have been led to a very different conclusion; with me it has occurred very often, and more than once with a violence to threaten dissolution. I am fully satisfied that were patients subjected to the proper mode of examination, there would be no lack of cases of spinal irritation. The disease being seated in one part, and the symptoms appearing in another, is probably the reason why it is so often overlooked, and of course maltreated. I am well aware that in the following cases there is nothing but what has been ably illustrated by Dr. Thomas Brown, and also by other writers. So impressed am I, however, with the importance of the subject, that were the perusal of them to arouse the attention of a single individual to this class of complaints, I would account them not written in vain. I propose merely to select a considerable list of cases, all of which occurred in females excepting the first.

*Case I.*—3d April, 1826. J. H. Weaver, aged 49, of shattered constitution. Complains of dull pain at breast, with incessant cough, almost preventing sleep, copious muco-purulent expectoration, dyspnœa, palpitation of heart, headache, and profuse nocturnal sweats; pulse 95, bowels confined. Has been ill four months, and

---

* To elucidate the subject of Cholera still farther, a chart of the principal towns and countries, traversed in its geographical progress, accompanies this paper in the Englishman's Magazine. We regret not being able to present it to our readers.

treated with blisters to the breast, and cough mixtures, without benefit. Dorsal vertebræ, about 6th, 7th, and 8th, are painful on pressure; pain stretching forward to breast, so acutely as to cause him to cry out. He had his bowels freely opened with purgative medicine, was confined to the horizontal position, and a blister was applied over pained part.

8th April. Blister, after several applications, discharges freely, but has produced a good deal of febrile excitement, which is subsiding.

12th. Blistered surfaces healed, and all the symptoms mitigated; pain in spine confined to one spot. The blister repeated and kept open about eight days, restored him to his usual good health.

This patient had been given up for consumption, and certainly he bore some marks of phthisis. It appeared, however, to be merely chronic bronchitis, combined with spinal irritation.

*Case II.*—22d June, 1828. H. B., aged 21, a woman of stout habit, has been subject to cough for several years; for eight or nine months has complained of pain in right side of chest, nearly constant, increased on inspiration and coughing; weight and oppression at breast, difficulty of breathing, dry convulsive cough, occasional headache, and dulness of spirits. Pulse 90, full, tongue moist, bowels natural, catamenia regular. Was bled to 12 ounces, and had a small blister applied to the breast with very little relief.

3d July. Symptoms worse. The third, fourth, and fifth dorsal vertebræ are tender on pressure, particularly on right side, pain stretching acutely forward, along the course of the intercostal nerves, to pained part inside of chest, causing dreadful convulsive coughing.

The horizontal position was strictly enjoined, and 12 leeches ordered to pained part of spine, which gave immediate relief; this was followed by a small blister, kept open for a few days.

10th July. Expresses herself greatly relieved, cough and other symptoms nearly gone—and three leeches more completed the cure. About a year and a half afterwards, this girl being attacked with smallpox, the same symptoms recurred, but subsided with the fever.

*Case III.*—5th June, 1829. Mrs. A., aged 33, mother of six children, of delicate constitution, complains of intense pain of right side of head, which appears a little swollen, dry cough, pain and oppression at breast, little increased on deep inspiration; respiration hurried and laborious, pain and numbness about shoulders, stretching down arms, palpitation of heart, great debility, is fatigued on the slightest exertion, or even speaking, pulse 112, weak and irritable, bowels costive. Upper dorsal and lower cervical vertebræ are painful on pressure, most severe about third and fourth dorsal; pressure aggravating symptoms. Had a child about three months ago, and did not recover well; three weeks afterwards, was affected with violent pain at breast, for which she was three times bled, and as often blistered, with but partial relief. Has consulted three medical men, who uniformly recommended blistering the breast. Is much reduced in body, and considered by herself and friends to be consumptive. Three days previous to seeing her, she had come fifty miles by land and water for the benefit of sea air, and had caught a cold on her passage, to which she attributes the aggravation of her complaints. Day before this, had, of her own accord, applied 12 leeches to side of head without benefit. She had two blue pills at bedtime, followed in the morning by a full dose of salts and senna, which procured copious evacuations with abatement of headache and febrile symptoms. Was ordered 12 leeches to upper part of dorsal vertebræ, to be followed by a blister. As she lived a considerable distance from me, it was ten days before I again saw her. She was now so much better, that she was able to walk about, without fatigue; appetite and strength improving every day. Is still suckling her child; says she has not enjoyed such health since delivery, and describes her feelings after leeching as if something were awanting about her breast; blistered surface has discharged well, but is now healed. Pain in spine confined to between third and fourth dorsal vertebræ, and much easier. Nine leeches and a small blister about the size of a crown-piece, kept open about eight days, removed her whole train of symptoms. I saw her about four months afterwards, with slight return of same complaint, which was easily cured by the same treatment. In this case the horizontal position was only enjoined a few days at first. In some cases the horizontal position is a *sine qua non* in the treatment; in others it is by no means essential.

*Case IV.*—28th August, 1829. I was called to Mrs. B., aged 20, of delicate habit, who said that she was ashamed to see me, as she could not tell what she had to complain of only she felt weak, and her appetite was gone. Pulse 80, feeble, tongue moist, bowels natural, catamenia regular; stoops much, body reduced to a skeleton, so dull in spirits that she can scarcely be roused to the least exertion. On strict interrogation, admits having a slight feeling of weakness or weariness at breast. Upper dorsal vertebræ are tender on pressure, most about the fourth on left side; right quite free from pain, pressure aggravating symptoms at breast. About six weeks ago, after assisting the maids a short time at washing, her hands and forearms became covered with a florid eruption, which soon disappeared, and was succeeded by a slight cough and uneasiness about chest, which have since gradually worn away. Was treated with a solution of tart. antim., bark, and other tonics, but without effect. Six leeches were ordered to pained part of spine, which produced immediate relief. She was now sensible she had been laboring under more oppression at breast than she had been aware of. A small blister produced such constitutional derangement, and aggravated the symptoms so much, that I did not think of reapplying it. A few leeches were applied every second day for a while, making in all 31, which along with the horizontal position, greater part of the day, effected complete recovery. During the application of the leeches she uniformly felt herself getting better, symptoms returning a little before next application, which gradually wore off towards the end. In less than four weeks, her health and strength were completely restored.

*Case V.*—M. C., aged 23. In summer, 1826, I attended this girl in fever. She was advanced in the disease, and had been neglected before I saw her; was treated with local bleeding, blistering, emetics, purgatives, &c., as symptoms indicated. Her recovery was slow, and accompanied with a host of nervous and hysterical symptoms, which have continued more or less ever since. About two years ago the abdomen getting enormously distended, and communicating a doughy feel to the fingers, there was little reason to doubt that her bowels were loaded with feculent matter. A course of purgative medicine was ordered, which brought away a prodigious quantity of dark pitchy-looking fæces, mixed with mucus and slimy matter. The belly, however, continued nearly of the same size, but a little softer; the stools were less in quantity, but much the same in appearance. Her strength getting exhausted, and her faith having failed her, I was obliged to abandon the practice. About six months after this she complained of pain in right hypochondriac region, aggravated on pressure, with frequent attacks of bilious vomiting; upon questioning her, she admitted having pain about shoulders, particularly on right side. Several medical gentlemen saw her, and she was more than once blistered over the region of the liver, and salivated with mercury, to no purpose.

On the 18th Dec. 1829, I was again called to see her. She had been getting worse for some months, and is now confined to bed. Complains of pain and giddiness of head, pain and numbness about shoulders and arms, particularly right arm, dull pain over region of liver and abdomen, most acute about caput coli, occasionally stretching down thighs. Is much harassed with vomiting of acrid bile, eyes weak, speech has been hesitating for some months, is worse of late, stops often in the middle of words, abdomen reduced to natural size and feel, bowels open. Pulse 80, weak, menses have made their appearance all along, but a little irregularly. Says she is pretty easy while lying in the horizontal position, but all her symptoms are aggravated on getting up; gets so faintish in the erect position, that she is soon obliged to lie down. These symptoms led me to suspect the spine to be in fault. It was accordingly examined, and found tender throughout its whole extent; but particularly the cervical, lower dorsal, and middle of lumbar vertebræ. Pressure or the application of a sponge dipped in hot water, on the lumbar vertebræ, gave pain, aggravating pain in abdomen, and particularly at caput coli, pain shooting down thighs along the course of crural nerves. On pressing the inferior dorsal, pain stretches forward to right hypochondriac region, which she describes as distinctly the pain she has so long felt there; pressure on the inferior cervical produced a feeling of pain and numbness about shoulders, stretching down right arm, which has not had proper feeling for some months. But the most remarkable symptom of all, is in the upper cervical; slight pressure there increases the shooting pain above the head, and causes a feeling of constriction about the throat, increasing the impediment in speech, and causing difficulty of respiration. When the pressure is increased the pain becomes intolerable, the function of voice ceases, and the respiration is as completely stopped as if she were suspended by a rope round the neck. Whatever part of the spine was pressed on, pain was felt shooting along the course of the nerves, but most severe on the right side. The upper cervical and inferior dorsal were the two points most severely affected, and from which I judged it not unlikely the pain might spread along the spine: these I resolved first to attack. Six leeches were applied to upper cervical, and same number to lower dorsal vertebræ. These were repeated with relief, and two small blisters were afterwards applied. In five days, when I again visited her, I found that the leeches had bled very freely, and had produced considerable debility; her face was pale and blanched, and she could with difficulty turn in bed. The blistered surfaces discharged for about a fortnight. It was a month before she gathered much strength; these symptoms, however, were mitigated, and she spoke more freely. By the middle of April she was able to be out of bed the greater part of the day, spoke without hesitation, and was nearly free from former symptoms, but dorsal vertebræ, between 9th and 10th, were still a little tender. By the middle of June she could take exercise out of doors, had a good appetite, and the spine was sound except between the 9th and 10th dorsal vertebræ, where there still was tenderness on pressure, shooting through to right side, in which she still felt some uneasiness. Considered herself in better health than at any time since attack of fever.

The horizontal position may have been a good adjunct here, but that it was essential to the cure does not appear, as she was obliged to keep it most of the time for near six weeks before the treatment commenced, notwithstanding which she became every day worse. That the origin of the nervous system was in fault since fever, I doubt not, and that timely detection and timely treatment might have saved her from nearly four years' suffering and misery, and preserved her constitution from a shock from which it can never fairly rally, I as little doubt. The pain in side and shoulders, and vomiting of acrid bile, were certainly symptoms of inflammation of the liver, but it is plain it was merely suffering in function, from disease of its nerves, as the heart and stomach are often known to do from the same cause.

*Case VI.*—A few weeks ago I was called to see a young woman 21 years of age, whose prominent symptom was vomiting of everything she took. She had pain in right hypochondriac region, increased on pressure, and pains about shoulders, shooting down right arm, which she describes as stitches. Had a child in the 16th year of her age, from which she dates the commencement of pain in side; pain in shoulders more recent, dyspepsia of some years' standing, but vomiting has only been distressing of late. Has been often bled and blistered for pain in side, (supposed to be hepatitis,) and sometimes with partial relief. Had consulted a medical practitioner a few days ago, who ordered a large blister to be applied over region of liver. Ninth and 10th dorsal, and 4th 5th and 6th cervical vertebræ, are painful on pressure, the pain stretching to pained part in side and shoulders. Nine leeches were immediately applied to 9th and 10th dorsal vertebræ, and in a few days same number to inferior cervical. Eight days after this she came a distance of about four miles, to show me how much improved she was. Vomiting gone, pain in shoulders and side much better, lies in bed most easily on right side, which she has not been able to do since she had the child; pained parts in spine still a little tender. Leeches ordered to be reapplied. I saw her about a week ago, stout in body and looking well. Says that she enjoys excellent health, to which she has been a stranger for more than five years. The horizontal position was not observed in this case.

I have only met with one case of this kind, which defied remedial measures; the prominent symptom was tickling cough; time, however, effected the cure. Several cases have been relieved, although they could not be said to be cured. This disease sometimes accompanies consumption, yet in one case I had strong reasons to believe that it roused up fatal tubercular phthisis.

That this class of complaints is seldom seen, except in the debilitated walks of life, appears to be unfounded. Anything here, in place of a town, scarcely deserves the name of a village. My practice is entirely in the country, in a place, too, famous for the salubrity of its air, and the healthiness of its inhabitants; yet in such a place spinal irritation holds no inconsiderable rank in the catalogue of human calamities.

As a stimulus to the younger candidate for medical eminence, I may be allowed to mention, that in the diagnosis and treatment of no other disease I gained so much credit and confidence in families. I have cured several who have long been considered to be falling victims to consumption, the gaunt and unrelenting destroyer of mankind. Restored to the arms of their families and friends from a long period of hopeless sufferings, they often know not in what terms to express their gratitude.—*Glasgow Medical Journal.*

3. *Cases of Pulmonary Consumption, with Observations.* By Thomas Henderson, M. D., Professor of the Theory and Practice of Medicine in the Columbian College, D. C.—*Case I.*—The subject of this case is a young lady, whose mother died of pulmonary consumption. The patient is sixteen years of age. It has been observed that consumption preys not "on the thorns and brambles of this wilderness, but on the rose and passion-flower of human excellence and gentleness." This was exemplified in the case of this lady. Apprehensive from her fragile delicacy of constitution that she would have phthisis, and attracted by her meek, gentle disposition, and by her personal loveliness, her friends watched over her with a tenderness that sheltered her from every impression which could hazard either emotion of mind or irritation of body. She was nurtured with all the vigilance and care required by the loveliest and most tender flower. Pulmonary irritation, notwithstanding, became evident, and this but redoubled the sedulous attention of her friends. She kept her room, avoided exertion, had a long course of medical treatment. In her case there was no amelioration: it proceeded steadily to a fatal termination.

*Case II.*—Miss ——, sister to the lady whose case has just been given, was for many years threatened with consumption. She had a very flat chest, cough, expectoration, great debility, pain in the side and breast, amenorrhœa, flour albus. She became much emaciated, and all who knew her were assured that the hereditary family disease would soon terminate her existence. Nothing that affectionate solicitude could procure was withheld. I attended her for a long time, endeavoring, while I guarded against inflammatory action in the lungs, to prevent the increase of debility. Particular attention was given to avoid active exercise or exposure to cold. Her health did not improve. Being sincerely pious, and desirous to practice what she professed, she insisted on attending evening prayer meetings, and on uniting with other institutions for benevolent purposes. These involved a change in her habits, *against which I remonstrated*, and in which she was opposed by her friends. She, however, gradually adopted her own course; took no medicine; went out at night, and exerted herself far beyond what was thought discreet. She improved in health; her mind became more cheerful; it was obvious that the change of habits had no unfavorable influence. The pulmonary symptoms declined. She married soon after, has children, and now enjoys good health, with every prospect of long life.

*Case III.*—Mr. ——, is the brother of the cases just related. He inherited a feeble, delicate frame, with striking resemblance to his mother, who died of phthisis. At eighteen he had a cough and pulmonary symptoms; but he entirely disregarded the monitions of hereditary predisposition, as actual symptoms of breast complaint, as well as what might be anticipated from a very narrow chest, tall and slender frame. He was engaged in laborious duties in a dry-goods store; afterwards pursued a life of much more active exercise, and exposure to all weather. He went two voyages to sea, and since follows mercantile business that requires bodily and mental activity. He preserved a good state of health for more than ten years. Of late he has had one attack of hæmoptysis; but except for the time has not confined himself, and is recovering well from it.

*Case IV.*—Miss ——, aged twenty, came under my care with many symptoms of tuberculous consumption, which disease had proved fatal to several members of her mother's family. My patient has hard cough, slight pain in the chest, expectoration of tough ash-colored mucus, great debility, pain in the loins, and there was an irritable, frequent pulse. I was exceedingly apprehensive of the result, and treated her case circumspectly in the way usually pursued by the best practitioners. She took digitalis cicuta; was moderately bled, was blistered, and all this without benefit. Seeing that no relief was afforded by medicine, and that the prescribed cautions as to exposure and exertion, denied her many of the enjoyments that a fine mind and cheerful spirit derived from social intercourse in visiting her friends, she gradually ceased consulting me, adopted her usual habits, and lost no health by giving up medicine. She travelled, married some time after, is the mother of several children, has pursued a very active life, and has no reason to apprehend a return of her symptoms.

The four persons whose cases have been detailed, were near relations. The first case submitted most rigidly to what was medically advised; she was closely housed, took much medicine, and yet the symptoms were not palliated, nor the fatal result procrastinated. In the other instances, strong predisposition to consumption; nay, actual symptoms were averted and removed by a course utterly at variance with what the physician advised. I do not mean to say that the same course would have removed the disease in the first case, for there are too many cases inevitably mortal. It is sufficient that the result of the three favorable cases be borne in mind in reading the observations at the close of this paper.

*Case V.*—Dr. B——, while a student of medicine, attracted my attention. His family had been severely visited with tuberculous phthisis. Within a year two brothers and one sister died of it. He had pulmonary symptoms, pain in the breast, cough; once spit blood; and his friends deeply feared that he would fall a victim to consumption. His brothers and sister were attended carefully by an eminent physician, who pursued the routine thought best for such cases; he adapted that routine with great intelligence to the several cases. I was consulted in two of the cases; no benefit resulted from medical treatment. Dr. B—— was so situated as to be compelled to walk two miles every day to attend the medical lectures, and to return the same distance at evening. This he did with unvarying regularity, through all kinds of weather, and frequently remained in the dissecting room till late at night. Such was his ardor for the acquisition of medical knowledge, that though with the above symptoms and much debility, he exposed himself to weather cold and wet, and to fatigue that others shunned. Occasionally the pain in the chest and debility were alarming—but he took no notice of them, and continued habits that *I urged him to avoid.* He, thus predisposed, continued these habits for three winters, and now lives in finer health than he has ever enjoyed. His relations who died, carefully shunned exposure and exertion.

*Case VI.*—Some years since I was called to see a gentleman, about twenty-two years of age. His father died of tracheal and tuberculous phthisis, and my patient had for some time labored under cough, purulent expectoration, hoarseness, pain in the breast. He had fever, with partial night sweats. I could not doubt, from his symptoms and the attendant emaciation, that his disease was confirmed strumous consumption, and that it would soon terminate fatally. I prescribed various remedies, anti-hectics, mild tonics, bleeding twice for the pain, mucilaginous mixtures for the cough, blisters, &c. As the autumn approached, I endeavored to impress on his mind my views of what should be his winter arrangements; these views were founded on flannel and the fireside; particularly that he should avoid exposure to cold and wet weather. He promised obedience to my advice as to the use of medicine and exposure during the cold season. I paid great attention to his case and symptoms, but the treatment did him no good. As his duties required exposure, he finally became impatient; went out as I thought at great hazard; abandoned the use of medicine, and as he rapidly lost flesh, it seemed almost certain that he would not live long. I discontinued my visits, as he took nothing, and was imprudent. He would walk for miles in the coldest weather without a surtout, and without flannel. He never avoided wet walking, or exposure to rain or snow. He took what diet was most agreeable to him; and now after four years' continuation of these habits, he is in much better health. The pulmonary symptoms are comparatively slight, and he apprehends no bad consequences from them.

*Case VII.*—About two years since, at the close of winter, I was consulted by a gentleman who had pulmonary symptoms. He had cough, expectoration, pain in his breast, lost his flesh, his constitution was delicate, his frame slender, and his chest narrow and stooping. I blistered him, gave him cough mixtures, and confined him to his room until the weather became mild. As he did not recover, I advised him to go by sea to New York, to reside in the country, to take exercise freely, and to avoid medicine, unless it became obviously necessary. I had the pleasure to see my patient some time after, with his symptoms entirely removed. Medicine had no agency in his recovery.

*Case VIII.*—Mr. ——, aged fourteen, lost his mother with tuberculous phthisis. It deserves remark here, that his mother was one of a family of nine, every member of which family died of this phthisis. This youth was growing up with every appearance of the scrofulous aspect; strong symptoms of pulmonary disease came on; as cough, thoracic pain, and I am informed that he once had hæmoptysis. He was advised to adopt the life of a sailor, the laborious duties of a common sailor. He went out before the mast, and after having been two years at sea, seems to have a renovated constitution, and is free from the pulmonary symptoms that threatened his life.

*Case IX.*—Mr. J——, lost his mother with tuberculous consumption. Seven years ago he had measles, which, as they declined, developed pulmonary symptoms, cough, pain in the chest, expectoration of tough dark mucus. Ever since he has suffered with obvious symptoms of tuberculous consumption: during the winter they are distressing.

He has taken no medicine, is bled when the lungs seem to be irritated, and when they occasion pain—and has derived great benefit from issues. But he has pursued a regular plan of out-door exercise on horseback and in walking, in winter and in summer. He is now better than when first taken. He attends the medical lectures; dissects at late hours, and is confident that his habits have baffled the threatened inroads of a most formidable disease. He does not lose blood habitually—only when obvious local plethora demands it.

I have been induced to review my experience in the treatment of phthisis pulmonalis, by reading two papers by Dr. Parrish, in the North American Medical and Surgical Journal. I could have adduced several other instances, but the above cases appear to me sufficient to justify some very interesting practical reflections. These may be submitted in the form of queries.

1. What has been the result in those cases of tuberculous phthisis which have been subjected to deliberate and judicious medical treatment, *unconnected with free exercise in the open air at all seasons?*

The answer is, that my long and extensive experience does not afford one case of recovery.

2. Has the medical treatment, *without active exercise*, materially protracted life in any cases?

The answer is, that it is very doubtful whether one life has been materially protracted.

3. Have bleeding, digitalis, mercury, prussic acid, tartar emetic, confinement to stove rooms, blistering, emetics, cicuta, ipecacuanha, inhalations, liverwort, have any of these, *without active exercise*, arrested, averted, or cured one case?

Confining this answer to tuberculous phthisis and to my own experience, the answer is in the negative.

4. Has any one case been averted, arrested, or cured, the successful result in which has not been owing to exercise, exertion, to free exposure to weather at all seasons, in walking, riding, and sailing?

The answer is, that this course of habits seems to have been the only preventive or curative agent.

5. And yet another question recurs, viz., in reviewing my practice, is there not reason to believe that many remedies so much relied on, and so authoritatively commended, have been either positively or negatively injurious, if not both?

The answer to this question may be inferred from what follows. The subject should be cautiously investigated, but requires most serious consideration.

If the opinions and experience of Dr. Parrish and others, and if the cases just detailed prove anything, they demonstrate this, that many cases of phthisis that were in progress of medical treatment, and were doing badly, on discontinuing the medicine, and in relying on active exercise and plain nutritious diet, did well. It would appear that a continuation of medical treatment in other cases did no good; the disease terminating certainly, and often speedily in death. This was probably owing to the intractable nature of the disease preying on constitutions unable to withstand its ravages.

We should, however, inquire and reflect seriously, whether remedies, such as are used in consumption, if they be not beneficial, be not injurious. It is not probable that bleeding, mercury, digitalis, prussic acid, &c., will be negative in such a disease, and in such constitutions as the phthisical have. So uniform is their failure to cure, and so frequently are they injurious, that the routine of prescription is formal at best, and hazardous in many instances. This formality, this routinism, is the bane of improvement in our science; and Dr. Parrish deserves the thanks of the profession for the firmness and intelligence with which he has assailed it.

If it excite surprise when I say that not one recovery from phthisis has occurred in my experience, let it be observed that illusion is made to *tuberculous* consumption. Chronic bronchitis, catarrhal phthisis, and that from ordinary suppuration or abscess in the lungs, are not in view. I have often seen recoveries in these cases, and have reasonably ascribed the recovery to medical treatment. Not so with tubercular phthisis. In this fell disease, if a remedy there be, it is yet to be discovered; and he is a wise practitioner who avoids the lædentia in scrofulous consumption. This form of phthisis is equally fatal wherever it exists; and where is it not? If in the admission that the remedies used may have been positively injurious, there appear too much candor, it is replied, I have followed the best lights that have been supposed to gleam along the dark path of tuberculous phthisis.

Experience has long since taught me that the writings of Dr. Rush on pulmonary consumption are not so much to be relied on as his precepts and practice in many other cases. The peculiar action of tubercular disease in the lungs, arising as it does from strumous complication, sets at nought the simplifications of ingenious hypothesis, and much more the boldness of systematic practice. Consumption consists in something more than the action of chronic pneumony, and invariably baffles the depletory measures directed to reduce that action. The origin, increase, and softening of tubercles, involve more in practical consideration, than the mere question whether or not they are the result of inflammation. It is in the incipient stage of tuberculous phthisis, that the practice of Rush is to be deprecated. It is to this incipient stage that the plan revived by Dr. Parrish is applicable, though not a few cases are on record in which the plan was advantageously pursued in more advanced stages. One of my patients appeared to be in confirmed consumption, and yet has been apparently preserved by this hardening system.

There is one thing to be considered in the treatment of consumption, and it is very important. Take a patient threatened with this disease, or with it formed; confine him to his room; deny him active exertion; bleed him frequently; put him on low diet; prevent unreserved intercourse with his friends; give him mercury, digitalis, cicuta, hydrocianic acid, and you surround him with a gloom that has most positive influence on his nervous system. Nervous depression and irritation, derange, irritate, enfeeble vascular action, especially in the capillaries, and here is a superadded and exceedingly operative difficulty. It is vain to talk to patients about exercise with dumb bells, &c., in stove rooms. Patients lose their energy by the confinement, and many lose entirely their elasticity and hope, in the fear that this course of practice and seclusion arises from the belief that consumption is formed, and therefore incurable. In all chronic cases, mental energy and cheerfulness are conspicuously subsidiary in treatment; without them, our mercury, and foxglove, and bleeding, do harm rather than good. This system, then, of confinement, so impairs nervous energy, as to act unpropitiously on the bodily powers, on the capillary circulation in the abdomen, on the skin, and above all, in the lungs themselves, favoring obstructions and sluggish circulation there. I have heard a delicate female say, with great firmness, "if I have to live but two years, let me rather enjoy myself than be immured;" and, pursuing her own course, she lives and does well.

How different are the effects of accustomed exercise and habits on the nervous system, and by consequence, on general functional action. How exercise tends to equalize and invigorate capillary circulation, and thus to prevent local obstruction, and if used with judgment, to remove it when formed. Exposure to cold is supposed, in phthisical and other cases, to act unfavorably in two ways; on the skin generally by constricting it, exciting sympathetically the *irritatio introversa;* and secondly by direct impression on the air-cells of the lungs. The first mode of operation can be easily guarded against, by carefully adapting dress; while the injury from direct contact of air has always appeared to me to be hypothetical. Doubtless if a person habituated to stove rooms too suddenly inhales a cold atmosphere, it may be prejudicial; but this sudden change is not here considered or desired. An objection to exercise, is by some said to be, that it so hurries the circulation of blood through the lungs as to be prejudicial in tuberculous phthisis. Of this I have strong doubt. Too great exertion may do so; but this is not what we want the patient to make. On the contrary, moderate exercise regularly pursued on horseback or in a carriage, has, to my knowledge, reduced an irritable and frequent pulse in disease of the heart, to moderate and healthy action. I am sure that so far as exercise favors capillary circulation, it accomplishes an important object in the incipient, or even in the more advanced stages of phthisis.

It has been observed by Dr. Parrish, that the practice sanctioned by his cases and mine is not new. It is a practice which should be judiciously attempted and fairly tried by cautious perseverance. Let the patient keep the feet dry and warm; let him be so well clothed as to prevent sensations of chilliness; let him avoid those severities of weather that a man in health would shun; but after this let him take exercise in the open air, at first in the mode of gestation, then on horseback, and finally by walking. In winter let him go to a climate where there will be the least interruption to his active habits. In this case physic may be needed to remove painful incidental symptoms, and what this physic should be will be obvious. It may be that these habits, and the courses of remedies usually adopted, have been too much separated under the idea of their incompatibility.

In coming to the conclusions apparently justified by the above experience and reflections, and sanctioned by the excellent judgment of Dr. Parrish, I have had much to contend with. A physician should cautiously abandon practice sanctioned by authority, and should circumspectly enter on the field of experiment. Early education led me to rely much on medicine in phthisis. Facts, however, within my own experience, too numerous to be resisted, and too plain to be mistaken, have caused me much anxious consideration. These facts are submitted, as they may be useful. If duly estimated I think they will not be considered as overrated. We know much of the *pathology* of tuberculous phthisis, perhaps as much as we can expect to know. Bayle, Portal, Young, Read, Duncan, Southey, Rush, with many others, have illustrated every point within the range of consumption, *except the cure.* I have seen too many fatal cases, fatal after every effort that authority or reason sanctioned, to believe that we know any remedy that promises the least hope to the sufferer. What, then, shall the physician do who has thus been baffled in hundreds of cases? Let him listen to the candid intelligence and experienced judgment of Dr.

Parrish: let him test the practice, and confirm or confute the suggestion. Hippocrates sent his patients on foot to the walls of Megara—Sydenham sent one on a long journey to see a Doctor Robinson. In rhetorical figure and in laudable hope, the physician has said, that in the wilds of the unexplored west, at the base of the Rocky Mountains, the plant may grow, the flower may bloom, the root may penetrate that possesses the healing power over tuberculous consumption. If the views to be inferred from these imperfect remarks be correct, we may send our phthisical patients in pursuit of the expected blessing.—*Amer. Jour. of Med. Sciences.*

4. *Intermittent Hemiplegia.*—Dr. Elliotson has put on record, in one of his clinical lectures, a case of this kind, which is the first he has ever witnessed. Our readers are aware that we have recorded several cases of this description on our pages. We insert the present case for several reasons.

"There was a case of disease of the nervous system presented of a curious character, the first of the kind I ever met with—intermittent palsy. I have read of it in authors, and you will find it mentioned by Cullen as *paralysis intermittens.* Now among all the patients I have ever seen, and these amounted to between thirty and forty thousand, including those in various public establishments and private practice, I had never met with an instance of this description. It was a case of intermittent hemiplegia. The man was admitted into Jacob's Ward some time ago, and I mentioned his admission at the time. I gave him no medicine, because I was desirous of seeing whether his account was true or not. I seldom give medicine in aguish or intermittent complaints, till some one in the hospital has witnessed the occurrence of the paroxysms. He staid here three weeks without having a paroxysm. He was, however, a very respectable man, and I did not doubt his account. He then went out of the hospital, enjoined by me to return if his disease reappeared. One day when I came to the hospital some time afterwards, I found him in the courts, and he said he had been seized with a paroxysm that morning, and he actually was then in a state of hemiplegia of the left side. I saw it myself. I made him walk, and he dragged his leg in a semi-circular way, as patients usually do, when they are laboring under hemiplegia, and he could not raise his left arm. It began at ten o'clock, and this was the usual course of the disease. He had told me originally that the paroxysms came on at 10 o'clock in the morning, not every day, but every third or fourth day, and, with a single exception, never after a longer period than that; but, on one occasion, there was an interval of sixteen days. He was 48 years of age, and had been subject to this affection for two years and a half; and the paroxysm would last from three to four hours. But although it only lasted that time, he was not perfectly clear from it through the whole of the day. He never knew the paroxysms begin later than 11 o'clock, or earlier than 10; from 10 to 11 was the regular period till a week before he had been admitted, when one attack came on at half past ten in the evening—the usual hour, but in the evening instead of the morning.

The affection was not more frequent then than when it first began. The man looked sickly as if he had had ague, but still more as if he had suffered from a hot climate; and it appeared that he had been in the East and West Indies, and that he had had fever both at Bombay and Batavia. He had suffered from dysentary, and when he was in the hospital he had diarrhœa. I do not doubt that this was the effect of malaria—that his hemiplegia was a form of ague. I will not quarrel about words,—you might say it was not ague, because unattended with shivering, fever, or sweating; but I have no doubt it was as much the effect of malaria as ague is: it was merely a variety of the same affection of the system. Supposing this to be the case, and having witnessed a paroxysm myself, I now gave him the sulphate of quinine, and, as the disease was of long standing, I began with a good quantity,—five grains every six hours: this medicine very soon put a stop to the complaint, but not until I had increased the dose to ten grains every six hours, so that he took forty grains in the twenty-four hours. This is the dose that is often required in quartan ague, and the present was a worse form of the disease than the quartan, because it occurred on the third or fourth day; and the longer the attacks, the greater is the difficulty of curing the affection, which may be considered as so much the more of a chronic character. It is not a matter of wonder that that large quantity was required. He continued in the hospital from his first admission, on the 13th of October, till the 23d of December,—rather more than three months,—without any other attack whatever, and his health became greatly improved. It is wrong to suppose that malaria does nothing more than produce these particular forms of intermittent disease; it poisons the whole body, and many persons are destroyed by it who never had ague at all, so deadly is the poison. His health, however, regularly improved under the quinine; he became strong, his countenance was better, and altogether he found that he had received very great benefit. However, on the 28th of the same month, five days after his presentation, he came to me, saying that he had had a slight attack a very slight one, that morning, but still it was an attack, and it occurred rather later than usual, some little time after 11 o'clock. When I saw him, at about half-past one o'clock, it was then nearly gone off. I increased the quantity of sulphate of quinine to fifteen grains every six hours, and if that be not sufficient I shall give him more, as he is to come to me from time to time. I had a person in the hospital who was not cured of ague with less than a scruple every six hours, and therefore I shall not be surprised if that quantity be required in the case of this man; but I have no doubt that eventually he will be perfectly cured, though he may need very large doses.

"This is a very interesting case, proving that paralysis is not *necessarily* an *organic* affection; that hemiplegia does not necessarily arise from effusion, or from compression of any kind, at least of an organic nature. If any compression do occur in this man, it can only be during the fit, for at other times he is perfectly well; it is entirely, I presume, an affair of *function*, induced by a particular poison. I

have at this moment, in private practice, a very curious case, in which disease has arisen from malaria: it has occurred in a young gentleman about eleven years of age, who lives by the side of the Thames. He had diarrhœa at school, which was allowed to run on; he was, however, taken home, and treated very properly by the gentleman who attended the family, by leeches to the abdomen, and I believe a blister, and all went on very well. He had tenderness just on one side of the umbilicus; he was, however, seized, all at once, at a certain hour in the evening, with violent irritation, severe itching, tingling, and redness at the leech-bites, some feverishness, just at the very part where all the leeches had been applied, and every leech-bite became red and swollen. His sufferings were extreme, but, after lasting for a certain time, all these symptoms went away. At the same hour the following evening, the same thing occurred, the leech-bites became swelled and hot, and he fell into a state of general excitement, from, as it would appear, the itching and tingling. The medical gentleman immediately saw him, and thought the attack was of an aguish character; and, as the family lived in a low spot by the side of the Thames, he gave this lad twenty grains of sulphate of quinine, in divided doses, before the time of the next expected paroxysm. The attack came on the next evening, but at a later period than usual, showing that the remedy had produced an impression. It is common for the remedy not to stop the disease at once, but to cause the fits to be postponed. I, however, was sent for, and I told the family that I was quite satisfied that the youth was going on right; that the quinine was the only remedy, and that it must be persevered in at the same doses. The fits were very distressing—indeed, to the family, alarming, and we both agreed that it was better to go on with twenty grains in the twenty-four hours. The next day the paroxysms appeared later and more slightly, and then came on once in two or three days, and still more slightly; he presently became perfectly well. At the end of a month he went out of doors, and was exposed to cold, and from his extreme anxiety to regain the time he had lost from school, for he was a fine boy, a paroxysm came on again, but rather mildly; the medicine was again had recourse to, and the immediate effect was a postponement and alleviation of the next paroxysm, and I have no doubt that if he continue to take the remedy for some weeks, he will not have a relapse. These remedies will not cure the disease unless you give them for some time after the disease has appeared to cease. Sometimes it is necessary to give them for many weeks; sometimes it is necessary to do more than this—to remove the patient from the spot. Just as in syphilis, if a person get cured, and return to the same quarters, the mercury he has taken will, of course, not prevent him from again catching the disease; so a person may be cured of ague, but if he continue to live in the same unhealthy quarters, of course the poison may operate afresh upon him: and as, in syphilis, mercury must be taken for some time after all the symptoms have disappeared, so must quinine *after* ague."

We have seen many cases where paralysis was evidently not the effect of organic disease, but of malaria. One, which we are not at liberty to mention by name, but which we lately saw with Dr. Paris, (who will confirm the truth of this statement,) is still more remarkable than the case recorded by Dr. Elliotson. A young gentleman, the son of one of the most PUBLIC CHARACTERS in England, travelled, more enthusiastically than wisely, through Italy, during the summer of 1830. The consequence was, a malaria fever, after which he lost the power of *all* the voluntary muscles of the body, from head to foot! In this deplorable condition, he was conveyed from Venice to London, *like a corpse in perfect health.* All the functions went on regularly—intellectual and organic—but all animal power of motion and locomotion was lost. This general paralysis continued for some weeks after his arrival here; and Dr. Paris very judiciously abstained from violent remedies, such as strychnine, &c., trusting to the recuperative powers of Nature. Motion gradually returned—first in the upper, and afterwards in the lower extremities—and we believe he is now walking about, and enjoying good health. Such accidents, though seldom on so remarkable a scale, are every year happening in Italy and other malarious countries, though they are very imperfectly known to the profession of Great Britain.—*Medico-Chirurgical Review.*

5. *Fatuity from an accumulation of Ascarides.*—Elizabeth Whittingham, a girl of 14 years of age, of a pale, gloomy, and vacant countenance, whose employments were sedentary, and who had never properly menstruated, was admitted an out-patient of the Bath United Hospital, Tuesday, October 28, 1828.

Her appearance and manner gave the strongest impression of idiocy, or rather mania. The brow was contracted; the eyes restless, wandering, and betraying suspicion; and she made repeated attempts to escape from the room. She refused to answer, or did not comprehend the commonest questions, and betrayed the utmost reluctance to a protracted investigation. From the information of her mother, who accompanied her, it appeared that she suffered chiefly from drowsiness, pain of head, vertigo, syncope, and hiccup; that she had more than once fallen down, and was totally indifferent to everything, and every person around her; her abdomen was large and tense; she had flatus, borborygmi, and costive bowels; the pulse was quick and sharp; and the tongue slightly furred.

To be bled immediately to 10 ounces. To have a three-grain calomel-pill every other night, and a purgative draught every other morning.

31st.—Less drowsy and oppressed, but "memory entirely lost, calling one thing for another;" is freely purged, motions less dark, and has discharged a vast quantity of ascarides, collected and agglutinated into round masses; countenance and manner little altered.

Increase the dose of calomel to five grains every other night, and continue the cathartic mixture.

Nov. 4th.—Countenance dark and suspicious, but speaks rationally; the stools are more healthy, and fewer masses discharged.

Continue the medicine.

7th.—Countenance bright and cheerful, evacuations natural, expresses herself thankful for the attention shown her, and is free from complaint.

1829.—This girl, when last heard of, remained in good health, and actively employed in her usual business, that of a seamstress. Her case, is recorded, not on account of anything very remarkable being attached to it, but as capable, perhaps, of affording a hint as to prognosis. Her demeanor was so eccentric, and her whole appearance so decidedly maniacal, that it might, in some instances, have led to severe and improper measures of treatment or of coertion, and the unfortunate sufferer, it is possible, have been actually driven into that state, which was, at present, only simulated.

Nothing certainly could be more remarkable, or present a greater contrast, than the face of gloom in the commencement, and the cheerful and pleasing demeanor at the termination.—*Med. Gaz.*

6. *Mode of arresting Hemorrhage from Extraction of the Teeth.*—A very severe and dangerous hemorrhage occasionally follows the extraction of a tooth. That this is, in some cases, the result of a hemorrhagic tendency in the constitution—in other words, of a want of contractile power in the coats of the arteries, seems clear, from the circumstance that some persons never have a tooth extracted, without considerable hemorrhage continuing for a long time afterwards. Several cases are on record which have terminated fatally, notwithstanding every effort which was made to stop the flow of blood. The usual mode of treating these cases, is to roll up a small bit of lint in a conical form, and to press it into the bleeding alveolar cavity, and then to produce pressure by a compress placed over it. These plugs are often imbued with styptics of various kinds, and caustic is occasionally introduced into the alveolar cavity. Failing these remedies, the actual cautery has been repeatedly used, and even the carotid artery tied, without effect.

When it is recollected that the bleeding vessel is situated at the very bottom of the alveolar cavity, it is astonishing that the simple method of applying pressure in immediate contact with it, which I am about to recommend, should not long ago have been adopted. The plugs of lint which are usually employed, are so clumsy and thick that they cannot be forced half way down the socket; and even if they are made more nearly to fit the cavity, the pressure which can be made upon them is very imperfect and uncertain. I have seen many very severe cases, and some in which the patient had very nearly sunk from loss of blood; but I never saw one in which I failed to stop the hemorrhage by the following simple process.

The bleeding alveolus is first of all to be ascertained, the coagulum carefully removed from it, and the interior cleaned by a piece of lint, and by rinsing the mouth with warm water. A strip of lint of sufficient length being torn off, one extremity of it is then to be introduced by means of any curved instrument which will reach the bottom of the socket, and pressed down to the very extremity of the alveolar cavity; a further portion is then to be forced upon the first, and so on until the cavity is filled, and every part firmly consolidated, by each portion being pressed upon by the next above it: when the cavity is thus filled, and the lint rises rather above the edge, a compress is laid upon it, so thick as to be pressed upon by the antagonist teeth. The mouth is then to be forcibly closed, so as to retain the plug in its place, and keep it constantly and very firmly pressed against the bottom of the alveolar cavity.—*Thomas Bell on the Teeth.*

7. *Lithotrity in Asia.*—Dr. Civiale communicated to the Academy of Sciences of Paris, a letter addressed to the Minister of Foreign Affairs, by the French Agent in Bagdad, announcing the performance in the latter city of the operation of lithotrity on twelve persons, by a German Surgeon named Martin, and all of whom were perfectly cured, except a child who was obliged to be cut in consequence of the great size of the stone.—*Gaz. Medicale.*

8. *Case in which a large Spoon was swallowed, and produced Ulceration of the Duodenum and fatal Peritonitis*—By Mr. Houston.—The body of a maniac who died with symptoms of *peritonitis*, in the Richmond Lunatic Asylum, was brought to the College of Surgeons for dissection. The peritoneum exhibited all the marks of violent inflammation, viz., copious effusion of lymph, and sero-purulent fluid, with extensive adhesions. A small quantity of the contents of the duodenum was found out of the bowel in the left hypochondre, and a large rusty iron spoon, eleven inches long, was found lying across the bowel, with the handle directed foremost and downward, where it had produced ulceration and perforation of the bowel, while the larger end was lodged in the pylorus, which, though dilated, was neither ulcerated nor broken. The spoon was bent in the centre, where it rested on the vertebræ; but whether it had this form previous to being swallowed, or had acquired it afterwards, it was impossible to ascertain. From the position of the spoon, Mr. Houston infers that it had not been swallowed accidentally in the transmission of food, but had been forced down the œsophagus by the insane patient.—*Ed. Med. and Surg. Journ.*

9. *The Human Voice.*—At a late sitting of the Paris Academy of Sciences, some interesting facts were disclosed in a paper by M. Bennati, on the Mechanism of the Voice. From his professional engagement of physician to the Italian Opera, he has had extensive opportunities of forwarding the object of his inquiries. He has observed that the voice is chiefly affected by the elongation of the uvula, which most persons who have had severe colds will readily understand; in some cases, that part of the human organ becomes so enlarged as to entirely prevent the issue of any sound louder than a whisper. The case of a lady was instanced, who entirely lost the use of her voice for several months, and was reduced to the necessity of writing all she wished to say; recourse was had in vain to several eminent physicians: she at last was advised to use *a gargle of strong alum water*, which COMPLETELY RESTORED HER VOICE. A permanent organic enlargement is not unfrequent, in which case M. Bennati has successfully employed cauterization of the uvula, and has, in some cases, increased the *timbre*, and even added two or three notes to the compass of the voice. Those who wish to benefit by such a curious discovery, will be glad to know how this is effected. He makes use of a metallic instrument, at the end of which is a bowl containing the lunar caustic, so shaped as to touch, simultaneously, the whole surface of the uvula, having a sliding lid to prevent contact with any other part, which is acted on through the handle of the instrument. The effect of the caustic is to excite the contraction of the muscles, and reduce the part to its ordinary dimensions. A few applications will prove its efficacy. An instance is cited, that of a pleader, who, after speaking a short time, lost the tone of his voice, his throat became dry, and convulsive coughing ensued; and he was obliged to relinquish pleading. Having consulted M. Bennati, it was discovered that the uvula was considerably elongated. He employed the caustic, and in nine applications his voice was completely restored, and he is now a distinguished advocate.—*London Athenæum.*

10. *Case of Poison by Rattlesnake.*—By Dr. Hiram B. Philips, of Buncombe County, North Carolina.—As I conceive that a record of cases is of the first importance to the science of medicine, I hesitate not in placing the following paper before the medical public. No originality is laid claim to; my sole object consists in corroborating the practice recommended by Mr. Ireland, by showing the beneficial use of arsenic against the poison of the rattlesnake. Without any further comment, I shall proceed to relate the case.

On the 25th of July, 1830, at 8 o'clock A. M., I was called to visit Miss Happy Briggs, who had been bitten twenty-six hours before by a rattlesnake. She had received two wouuds; one on the instep, and the other near the great toe of the left foot. Her body was considerably swollen; her eyes almost closed; tongue tumid; deglutition impeded to some degree, and articulation indistinct. Her left leg, as high as the hip, was enormously distended, and threatened mortification; the skin having a shining appearance, with discoloration, being black on the outside and mottled on the inside with black and yellow spots, so that one might have fancied it resembled the skin of the snake. The bitten part pains her severely; and the inguinal glands on that side were much enlarged; pulse low, and about 60 strokes in a minute, and surface cold. She felt extreme nausea, and vomited on making the least exertion. Great thirst was an attendant symptom from the first, so much so, that previous to my arrival she had allayed it with an immoderate quantity of water; her bowels were rather constipated. Her mind appeared not in the least affected.

I commenced my treatment by making longitudinal incisions from the left knee downwards to the feet, and scarifying the wounds, and then blistered the limb extensively. She took the volatile alkali in f. dr. ii. doses at intervals for two hours without any good effect. Finding the circumstances thus, I determined to have resort to arsenic, and Fowler's solution not being procurable at the moment, I employed the oxide, commencing in doses of a quarter of a grain every fifteen minutes, for about two hours, when I perceived a material alteration in my patient; the swelling diminished somewhat, the nausea ceased, and she was able to articulate distinctly. By continuing the treatment with the arsenic for two hours more, all dangerous symptoms disappeared. All her body except the left leg, (which, however, was lessened,) was completely unswollen. Towards night she complained of head-ache, and her pulse rose to about 130, strong, full, and hard, when I judged venesection expedient, and extracted ten ounces of blood, which relieved her. I prescribed then the oleum ricini to obviate constipation, and effected the intention. After leaving the blisters twenty-two hours on the limb, they were removed, and the sacks formed by the cuticle, contained from a pint and a half to a quart of liquid, of a dark greenish colour. The blistered surfaces were dressed in the usual way, and in three weeks my patient, by taking a gentle purgative every third day, was restored to her former health and vigour.

I have thus endeavoured to sketch a case which, from the small number of similar kinds on record, I trust will not be unserviceable.

11. *Medico-Legal Examinations of two cases of sudden death from wounds.*—By Mr. Alexander Watson.—We have already made liberal extracts from the July No. of our valued Edinburgh cotemporary. The following is lengthy, but the cases shew the importance of attention to the minutia of medical evidence. Their peculiar novelty also renders them more interesting.

The first case is that of Ann Rennie, or Pollock, who resided at Gifford Park, at the south side of Edinburgh, whose husband was tried in the High Court of Justiciary, 13th Feb., 1826, and was convicted of having murdered her, but contrived to strangle himself in jail previous to the day of his execution.

The body of this unfortunate woman was inspected by Mr. Newbigging and myself, on the 13th November, 1825. We were informed of her having died suddenly. She seemed to be about fifty years of age, of stout form, of very low rank, having lived in a small, dirty, ill-furnished house, having only some shavings and straw upon the floor, covered with a rug, for a bed. The clothes in contact with the private parts were stained with blood. No appearance of injury could be perceived on any part of the body externally. But upon separating the *labia pudendi*, a wound about an inch and a quarter in length was observed upon the inner side of the right nympha. This wound was evidently recent, its surface being covered with coagulated blood. Externally, it consisted of a remarkably clean, straight incision, parallel with the nympha. Internally, the finger could be introduced in four different directions, to the depth of about 2 1-4 inches in each; upwards and backwards towards the division of the iliac artery; backwards towards the tuberosity of the ischium; laterally towards the hip-joint, and upwards towards the *mons veneris.* In each direction the wound was of nearly the same diameter, which readily admitted the finger, and had distinctly an obtuse termination. By injecting warm water into the large vessels, we found that none of them had been wounded, and the penetrating instrument seemed to have been forced only through the cellular tissue. The weapon had passed up to the peritoneum at the right side of the pelvis, under which there was a considerable effusion of blood, but had not penetrated it. Another very small, but also very clean external incision was observed at the side of that above described.

The cavities of the cranium, thorax, and abdomen, were each examined, and their contents found to be quite healthy and natural.—The hemorrhage, therefore, which had taken place from the wound, was the only cause which we could assign for her death; and this, we knew from the nature and structure (erectile spongy tissue) of the parts cut, must have been profuse.

Respecting the probable instrument with which this wound had been inflicted, it was obvious from the cleanness of the cut, that the external part of the incision had been made with a remarkably sharp-edged instrument; and from the very obtuse termination of each of the internal wounds, their small depth, and the absence of any injury of the important neighboring parts, it was highly probable, that tho' the instrument that inflicted the wound must have had a very sharp edge, it had a round or blunt point. Now, the only very sharp-edged instrument which I conceived such persons were likely to have possessed, was a *razor.* This instrument has also a blunt point, and could not be inserted to a greater depth than two or three inches, on account of the fingers holding its blade in so using it. I also conceived, that after the external part was cut, any sort of knife could have been thrust into the parts, so as to have formed the wound above described. This I verified by experiment with a razor upon the dead body.

It was proved by evidence, at the trial of the husband for this horrid crime, that two old rusty table-knives had been found in the house, as also two *razors.* One of these razors had its blade and handle covered with blood, and was found concealed in a piece of green cloth. These particulars rendered it almost certain that this had been the weapon used; so that the nature of the wound previously pointed out the probable instrument with much accuracy; though the circumstance of such having been found in the house was then quite unknown to the medical witnesses.

In this case, two other points occurred for the consideration of the medical jurist. Could she have inflicted this wound herself? Several circumstances which appeared in evidence, rendered this impossible; 1*st.* She was in a state of intoxication at the time; 2*d.* Her husband was in the house along with her,—saw her state,—went for a surgeon, (Mr. George White,) and never alleged this at the time; 3*d.* No lethal weapon was found near her; 4*th.* The *pu-*

*dendum* was a very extraordinary place for a suicide to inflict a wound.

It was also alleged and plead for the prisoner, that she had fallen upon a piece of broken earthen ware and cut herself. Could this wound, then, have been occasioned by such an accident? It was impossible to conceive that any broken piece of earthen ware in common use could, by the woman accidentally sitting down upon it, have inflicted such a clean external incision, and produced the wound in the different directions above described. Such a portion of earthenware, if sharp pointed and advantageously placed, might certainly have produced a pretty severe wound of the part; but it would have been a *lacerated* wound, not of any great depth, and not larger internally than externally, and in no part greater than the size of the wounding body; and it was very improbable that it would have had different directions internally.

The second case, which is very similar to that just detailed, is that of Mrs. Bridget Calderhead, who resided at Dunbar Street, near the Canal Basin, and whose sudden death was occasioned by a wound received on the morning of the 1st January, 1831. Mr. Mitchelhill, surgeon, had been called in to see this unfortunate woman soon after the accident, but found her dead when he arrived. At the request of the Sheriff, I accompanied Mr. Mitchelhill in the examination of her body, which we performed on the afternoon of the 1st January, in the Police-Office, to which place her body had been carried.

We found the body of this woman dressed in her ordinary day clothes, and covered with a blanket. We removed her clothes very carefully. They consisted chiefly of a printed cotton gown, two flannel petticoats, (one blue, the other white,) and a shift. These seemed almost quite new, and had no wound or tear in any of them, with the exception of the blue petticoat, in which there were some small worn holes. The lower part of all these garments had been drenched with blood, with which they were still wet.

We then discovered that the hemorrhage had proceeded from a wound upon the middle of the left *labium pudendi.* Externally, this wound consisted of a very clean incision about three quarters of an inch in length, having a straight direction parallel with the margin of the *labium.* When the finger was introduced into this wound, it entered into a bloody cavity, sufficient to have contained a small hen's egg; and from this cavity the finger passed to a greater depth in three different directions,—viz: upwards towards the under part of the *symphysis pubis*,—downwards towards the *perineum*, and backwards by the side of the vagina and rectum. Its greatest depth at any of these parts was between two and three inches. When the internal part of the wound was laid open, the divided orifices of several pretty large arteries and veins were seen, and particularly the divided extremities of the large artery going to the clitoris. The orifices of these vessels, as well as the internal surface of the wound, had the appearance of having been very clean cut by a sharp instrument. I removed the wounded parts, and preserved them in spirits.

On the back part of the head there was the mark of a contusion, which had occasioned the extravasation of a small quantity of blood, upon the anterior surface of the brain. The cavities of the chest and abdomen were quite natural.

There could be no difficulty in this case in ascribing death to the excessive hemorrhage occasioned by the wound at the *labium pudendi.* We therefore gave this as our opinion as to the cause of death. The other question then arose, What was the probable instrument and manner in which the wound had been inflicted? From the straight, very clean incision externally, in length corresponding exactly to the breadth of the knives in most common use, as well as from the extent, cleanness of the wound internally, and its different directions, it appeared pretty evident, and most probable, that it had been inflicted by some kind of knife; and, indeed, it obviously could only have been produced by several thrusts of a knife in different directions. Some pieces of a broken wine-glass, however, had been found adjoining to some of the blood the woman had lost at the foot of the stair where she had received the wound. It therefore became a question of importance, whether or not the wound could have been occasioned by her having fallen upon these broken pieces of glass?

1. Let us consider, Is it *physically possible* that any of the portions of the broken wine-glass, or of any wine-glass in common use, could have made such a wound? A portion of glass capable of forming such a wound must have been between two and three inches long, about three fourths of an inch broad, having a sharp cutting edge, and some degree of point; and it must also have possessed sufficient strength to permit its having been moved about and thrust in different directions, without breaking. To form such a piece out of a common wine-glass, is evidently beyond the utmost ingenuity of man; far less, then, could such a portion have been formed by accidental fractnre.

The broken portion of glass found, consisted of the stalk of a common wine-glass without the foot, having at its upper part the bottom of the cup of the glass, of about an inch in diameter, attached to it transversely. Nearly the whole of the cup of the glass was broken off, leaving only a few fragments of the sides projecting upwards from the bottom which remained. The part of the stock of the glass which remained, was about an inch or an inch and a half in length, and its lower part had been clean broken across, leaving no sharp point. It was, therefore, quite obvious that this fragment of glass was completely incapable of forming the wound in question; for, the length, form, and sharpness which were requisite, were completely wanting; and any wound which could have been occasioned by it, must have been quite different in its character. A wound from such a piece of broken glass would have been a *lacerated wound*, not a *clean incision*, not larger internally than externally, and could not have had several different directions internally. The upper end of the glass would have made several small lacerated wounds, while the lower end could not have made any wound at all. I therefore came to the conclusion, that it was physically impossible for the wound in question to have been produced by any portion of a broken wine-glass, such as that found.

2. But supposing that it had been physically possible for such a wound to have been occasioned by a fragment of broken glass, was it in any degree *probable* for it so to have happened in this particular case? In order to have accomplished this, the requisite portion of glass must have been standing ready to receive the fall of the wounded part upon it; the person must have either sat down, or fallen forward upon it, and her clothes, at the time, must have been completely out of the way, as there were none of them wounded; and moreover, the penetrating piece of glass must have thrust itself in different directions, making clean incisions internally as well as externally. So that a concurrence of circumstances highly improbable, nay, almost miraculous, would have been required, in order that the wound in question could have been occasioned accidentally.

From a careful consideration of the foregoing circumstances, I came to the conclusion, that it was neither physically nor morally possible that the wound received by this unfortunate woman, could have been caused accidentally by her having fallen on the portion of wine-glass alluded to; and such, I believe, were also the opinions of Mr. Mitchelhill, and of Dr. Christison, who gave his opinion on the case at the trial. But it could not be denied *to be within the verge of possibility*, that the wound might have been occasioned by her having fallen upon some other piece of glass or sharp body, and therefore we each, individually, thought proper in giving our evidence at the trial, to qualify our opinion upon this point, by saying that we considered it to be *scarcely possible, and very improbable.*

I was also asked at the trial, whether or not this wound could have been produced by her having fallen accidentally on a pair of scissors; to which I stated, that I did not think it could by any of the scissors in common use. For it is obvious that, to have produced such a wound, the blade or blades of the scissors, when together, must have had the sharpness of a knife, as also the requisite length and breadth; besides being in an erect position when fallen upon, and afterwards moved in different directions to form the internal wounds; a combination of circumstances which, as already mentioned respecting the glass, I conceived to be nearly impossible. I may add, that neither scissors nor pockets were found about the person or clothes of the woman.

By the evidence adduced at the trial of two young men, brothers, of the name of Duncan, for the murder of this woman, it was obvious she had received a wound at the first floor of a common stair, almost immediately after which, she was precipitated headlong to the bottom of the stair. When she was there found, upon being set up, blood was observed trickling down her legs. Much stress was laid by the counsel for the panels, upon there having been no blood seen upon the stair. But when it is recollected that she had on her two thick flannel petticoats, a gown and shift, which were each drenched with blood, it is obvious that the clothes, absorbing the blood when it first flowed, and her rapid progress head forward down the stair, easily explain this circumstance.

The above are two cases very similar in their principal features, and appear to me to be highly interesting, in a medico-legal point of view. From the details here given, it is obviously necessary, in such cases, for the medical jurist, to form an accurate opinion whether the death had been occasioned by violence or by natural causes; and, if by violence, to say whether most probably by accident, suicide, or murder. These form considerations of the highest importance to medical men, as upon their evidence the lives of others frequently depend.

In the above cases, it is also obvious, how very accurately the nature of the wound may point out the probable weapon of its infliction; and how very important this circumstance is, in establishing the criminal design with which such wounds may have been inflicted.

In both of the above cases, I removed and preserved the wounded parts, by cutting out along with them a considerable portion of the soft parts; at the same time carefully preserving entire the external wounds. This I consider to be a matter of considerable importance in all such cases; 1*st.* That the wounded parts may afterwards be examined more minutely; 2*dly.* That any lethal weapon or other sharp body afterwards found and supposed to have occasioned the wound, may be compared with it; and 3*dly* That the nature of the wound may be seen by any other medical jurist who may afterwards be consulted in the case, either by the crown or in behalf of the accused.

Before concluding, I shall make a few remarks on wounds inflicted in the situation of the cases just detailed. In both the above cases this part of the body seems to have been selected by the murderers to effect their design secretly. For in both, particularly in the first, the wound was concealed to a superficial observer. And they may have had an idea, from the frequency of "flooding" in females, that their deaths might have been supposed to have happened either from this cause, or by accidentally injuring themselves by sitting down upon some sharp body. For, it is a curious fact, that in both cases, the probable murderers* were the first to go for medical aid to the deceased.

Both cases also show, that wounds of the external parts of generation in the female may prove fatal by excessive hemorrhage; the peculiar structure of the parts consisting of erectile spongy tissue, which is very vascular, giving rise to a continued, steady, though not very rapid, flow of blood.

---

*Though the Duncan's got off by a verdict, by a plurality of the jury, of "not proven," yet there could be very little doubt of the guilt of one of them. It was impossible, however, to ascertain which, so that both were transported *for assault.*

In both cases the defence set up by the panel's counsel of the deceased having cut themselves by having accidentally sat down upon broken sharp bodies, was certainly plausible, and could only be controverted by the careful examination of the nature and form of the wounds, and a comparison of them with the alleged inflicting bodies. In both cases this was of great importance, as the deceased did not live to narrate the causes of their wounds; and in both, it appears to me, that the inferences deduced were beyond the possibility of doubt.—*Edin. Med. and Surgical Journal, July*, 1831.

12. *Case of the successful treatment of Snake-bite by cupping glasses.* (*Transactions of the Medical and Physical Society of Calcutta*, vol. iv.)—Mr. Clark, assistant surgeon in the Bengal service, has related the following instance of the successful application of Dr. Barry's treatment against the effects of poisons introduced into an external wound.

A fine healthy youth, fourteen years of age, was bitten at 10 6. M. near the bend of the right arm by a snake, the species of which could not be ascertained. He was immediately conveyed to a set of "Snake-Doctors," who, after practising their quackery for five hours to no purpose, directed him to be taken to a European surgeon. Mr. Clarke, who saw him immediately after the native doctors gave him up, found him affected with stupor, insensibility, laborious breathing, extreme feebleness and rapidity of the pulse, great expression of anxiety and distress, constant efforts to expel frothy matter from his throat, and inability to articulate. The wound was situated a little below the bifurcation of the vein at the bend of the arm, extended horizontally half an inch under the skin, and a slight elevation was perceptible over its course, which was paler than the surrounding integuments, so as to present the appearance of a splinter of fir under the skin. The adjacent integuments to the distance of two or three inches were tense and painful. The wound was immediately scarified, and a ligature applied above the elbow. At the same time two attempts were made to administer ammonia, but without success, as the muscles of deglutition were thrown into violent convulsive action, producing increased difficulty of breathing. Mr. Clarke then resolved to try the effects of cupping-glasses, and immediately applied one in the usual manner. After it had been applied for an hour and a half, with occasional short intervals, a manifest improvement had taken place. This was seven hours after the bite. He was more composed, and expectorated less; the pulse was fuller, and the sense of suffocation less urgent. The cupping-glass was now removed, as very little blood had been drawn by it; and ammonia was administered from time to time, but at first with difficulty. In the course of another hour he could stand up, and soon afterwards he was able to walk. Twelve hours after the bite, the bandage having been still kept on the arm, he complained only of drowsiness and difficulty in articulating. In twenty hours the drowsiness continued, the eyes were dull and injected like a person recovering from intoxication, and there was indistinctness of vision. The arm from which the ligature had been removed a short time before, was painful near the puncture, and on the seat of the ligature. Next day he was quite well, after a long and refreshing sleep.

13 *Culpable Abortion.** In the following case there are some facts of interest, and some points for criticism, notwithstanding the high rank which the reporter has attained in forensic medicine.

Messrs. Foderee and Ristelhueher having repaired to the house of the deceased, on the 7th March, 1822, they found the body of Catharine S. extended on a table, and already exhibiting marks of incipient putrefaction, although death had only taken place the preceding day, after a short illness. On examination, there was nothing particular observable in the chest. In the abdomen, the peritoneum was found inflamed, and some spots of inflammation on the mucous membrane of the stomach and bowels. In the neighborhood of the uterus, there was a sanguineous effusion, with some clots of blood, in the midst of which was found a fœtus of about 60 days' growth, with its umbilical cord. The womb was flattened, red, and inflamed. On more minute examination, it was found to be ruptured, the opening being about the size of a three franc piece. The internal surface of the uterus was also inflamed. The membranes of the fœtus were found perforated in two places—near the cervix uteri, and opposite the rupture in the parietes of the uterus, through which the fœtus had escaped into the abdomen. Nothing remarkable about the external parts of generation.

The medical commission called before them and examined several witnesses, by which it appeared that a midwife had been closeted with the deceased a day or two previous to her death, and had made use of a syringe with a long ivory pipe, which syringe was produced. Shortly after this interview, the deceased discharged a quantity of black blood from the vagina, and was seized with excruciating pains, which continued till death took place. From these data the Reporters came to the following conclusions:—viz. 1*st.* That the deceased died of a violent inflammation with rupture of the uterus, followed by expulsion of the fœtus into the abdomen. 2*dly.* That this inflammation and rupture being rare accidents, and not likely to take place, except as the effects or consequences of a grave malady, in the last stage of utero-gestation, it was probable that they were, in this instance, the result of violence, from the forcible introduction of the pipe of a syringe into the os uteri, and the injection of some acrid and stimulating liquid into the cavity of that organ.

The jury found the midwife guilty, and condemned her to ten years' imprisonment.

Some strictures are made by the French Journalists on the testimony of Foderee. They observe that the medical man has no right to call or examine witnesses, in cases of this kind. He should confine

---

*Rapport sur une provocation d'Avortement par M. Foderee. Gazette de Sante, November, 1824.

himself to a rigorous examination of the body, aided by the light of physiology and pathology, leaving all matters of moral evidence to the investigation of the civil magistrate. This may be a good general rule; but we think there are many exceptions to it. For instance, in the above case, had no information of instrumental interference been obtained, there was nothing in the body which could have authorised the surgeon to pronounce upon such interference. The rupture of the uterus was not considered as resulting from the operation of the instrument, but from the contractile efforts of the organ itself—and the rupture of the membranes opposite the mouth of the womb might surely proceed from the same cause. It was, therefore, the moral evidence that decided the question. We read lately of a case of spontaneous rupture of the uterus, at an early period of utero-gestation, though we cannot recollect where. This early period, therefore, is no proof of criminal interference, though, backed by the moral evidence, it becomes a very strong presumptive one in the present instance.

The case is otherwise instructive, as showing the danger of procuring abortion by criminal interference.

14. *Hæmatemesis cured by Emetics.** The practice of giving emetics in vomiting of blood is not new—but it is very rarely put in force. There is something repugnant to the feelings, or at least to the preconceptions, in giving an emetic to a man vomiting or spitting up quantities of blood. But we are, as yet, very imperfectly acquainted with the modus operandi—or even the *effects* of medicines on the human frame. Many years ago we saw a surgeon, by no means well versed either in therapeutics or pathology, exhibit a scruple of the sulphate of zinc to a man who had vomited up nearly a chamber-potful of blood, and who appeared nearly in articulo mortis. The patient vomited twice or thrice after the medicine was exhibited—but little or no blood appeared in the ejected matters, and he quickly recovered. We confess that we have not had courage enough to pursue the same practice since.

Dr. Sheridan informs us, that his father, who practised medicine with repute in the county of Cavan upwards of half a century ago, was in the habit of giving emetics of ipecacuan in cases of hæmatemesis, and that they never disappointed his expectations. Dr. Sheridan, the younger, was deterred from following his father's practice for many years; but having witnessed some fatal cases of the disease in question, he at length ventured on the remedy, and the present paper contains the particulars of five cases successfully treated. We shall give a brief outline of two or three of these cases, as they may inspire confidence in the treatment of a formidable disease.

*Case* 1. Jane Halpen, a young woman, complained of great oppression and pain about the præcordia, attended with violent palpitations.

---

*Dr. Edward Sheridan. Dublin Trans, vol. iv.

When our author first saw her, she had thrown up much dark coloured blood; but the vomiting had stopped, though the præcordial oppression continued. He did not, therefore, give the emetic. On the third day, the hæmatemesis returned with violence, and was followed by great debility. On the third day from the last mentioned attack, it recurred for the third time, and was excessive in quantity. He now exhibited thirty grains of ipecacuan. The vomiting ceased for a few minutes, but soon came on again, and blood was discharged, both grumous and fluid. She now lay in a state of the most alarming debility. No more blood issued—dropsy supervened, and was removed by aperients and diuretics.

*Case* 2. A builder, much exposed to the atmospheric vicissitudes, had not enjoyed good health for ten years past. He was subject to much pain and oppression about the præcordia. On the 24th December, he was seized with hæmatemesis to a great extent, attended with much palpitation, and pain across the præcordia. He had ejected more than three quarts of blood, when our author ordered an ipecacuan emetic. It rested some minutes on the stomach, and then brought off about a pint of blood, at three or four operations. "Here all the symptoms subsided: he found himself perfectly relieved: heat was gradually diffused over his whole surface, which had been cold before. He fell asleep: gentle perspiration ensued, which continued a good part of the next day: his pulse became full, soft, and regular, and he felt himself, he said, more free from pain, oppression, or sickness, than he had been for years before. On the day after, I gave him a mercurial purge, which brought away a great quantity of such matter as he had vomited, with this difference, that it was blacker, and resembled the discharges in melæna. He continued to improve, but was still weak: in this state, contrary to the most positive injunctions, he made too free, and the pain across the præcordia, the oppression, &c., returned. Under these circumstances I did not hesitate to repeat the ipecacuan: all these symptoms vanished, and he since continues to improve."

*Case* 3. This was a relapse of the first case, after an interval of three years. When our author arrived, the poor woman had vomited more than three quarts of blood. Her anxiety was extreme—pulse scarcely perceptible—palpitation great. With difficulty an emetic was administered. After taking it, her stomach became quiet for ten minutes. She then threw up a quantity of watery and mucous fluid, but no blood. "Immediately after, all the symptoms, as if by magic, ceased."

"These cases," says our author, "with the two others which I observed in the country, have made such an impression on me, that I never can hereafter hesitate for a moment to have recourse to the ipecacuan emetic in hæmatemesis. I do not mean by this term a mere vomiting of blood, which may be produced by various causes, and in some cases of which emetics might prove very prejudicial, but the true hæmatemesis, which I think will always be sufficiently distin guished by the symptoms which have been above described."—*Medico-Chirurgical Review.*

15 *Recto-vesical Lithotomy.*—This operation was first practiced in France, where it has been very coldly received. It was imported into Italy, and there it seems to take root and thrive. It has been practiced at Turin, Genoa, Milan, Pisa, but in different modes. Some surgeons have abandoned it after trial; others have written against it on theoretical grounds, and without trial; while the great number who have performed it, give it the preference to either he lateral or the high operation. There are some solid and important facts connected with this subject, which are not undeserving of attention. The following are some of them. Of 69 who have undergone the recto-vesical operation, 13 have died, or rather less than one in five. Of these 13, seven are asserted to have died of affections totally independent of the operation, 56 have been cured; but out of these 56, there are eight with fistulous openings. Of these eight, one had the lower part of the bladder (le bas-fond de la vessie) incised. Forty-eight therefore have been cured without fistula, in four of which the "bas-fond" had also been injured. The period of recovery varied from ten or twelve days to seven months. Seven were cured between the eighth and fifteenth day; twenty-eight between the fifteenth and thirteenth day; ten between the 30th and 60th day; and eleven between the 60th day and seven months.

The principal advantages of the recto-vesical operation are considered to be—the impossibility of wounding the pudic artery—the absence of hæmorrhage during and subsequent to the operation—the facility of extracting the largest calculi—the little risk of urinous infiltrations. On the other hand, the serious inconveniences of this operation are candidly stated, and are as follows:—the wounding of the seminal vessels—and the chance of recto-vesical fistula. It is yet to be proved that the first accident entails impotence.* In respect to the latter, it is said that it will be very rare where the incision does not go beyond the limits of the prostate gland. It will be frequent, but not inevitable in those cases where the incision reaches the lower part (bas-fond) of the bladder. In the lateral operation, there is little danger of either fistula or wound of the seminal vessels; but then there are other dangers and inconveniences, viz: 1st, Injury of the rectum—an accident by no means extremely rare, even in the hands of the most expert operators. 2d, Hæmorrhage, examples of which are, unfortunately, but too frequent; and generally mortal where the pudic artery is divided. 3d, The impossibility of extracting a very large stone by the lateral operation. 4th, The greater

* We know an instance where, after a successful lithotomy in the lateral way, the seminal vessels have been obliterated, and all communication with the urethra cut off. The inconveniences are rather distressing though the gentleman is upwards of seventy years of age. It is probable that inflammation about the verumontanum was the cause of this occlusion of the seminal vessels.—*Rev.*

mortality by the lateral, than by the recto-vesical operation. This parallel M. Breschet has drawn from the memoirs of Vacca and others, in Italy, and he comes to the conclusion that—the recto-vesical operation is, or appears to be, preferable to the lateral; and that it ought to be more frequently practiced than it now is in France.—*Arch. Gen. July*, 1824.

P. S. As many of the readers of this series may not be acquainted with the method of recto-vesical operation, we shall give a very short description of it in this place, in the words of a transatlantic writer, Dr. Muter, of New-York, formerly of Wisbeach, in Cambridgeshire, who has published a little work on the subject of Lithotomy, which does much credit to his judgment as an ingenious surgeon. There are two modes of performing this operation, one of which may be called the high, and the other the low recto-vesical incision. The former we believe is now seldom practiced. It consists in an incision made through the rectum and bladder, above the sphincter of the one, and prostate of the other, into the slit or groove of the staff, in the place where the bladder is punctured for suppression of urine; cutting a little to one side, to avoid wounding a small artery which runs along the middle of the anterior surface of the rectum.

"There is another situation," says Dr. Muter, "towards the verge of the anus and perineum, where a somewhat different operation may be performed, and a very large stone extracted. If I have maintained that a stone of small size only, ought to be extracted by the last, by this, I now assert that *only a large one* should be extracted."

"We are told by professor Vacca, that the fæces may be excluded from entering the bladder, by this mode of forming a valve of the rectum, opposite the wound in the prostate and neck of the bladder. The incision is to be made anterior to the prostate, and carried through the rectum, urethra, and sphincter of the anus, an inch into the perineum, cutting along the slit in the staff. The surgeon then runs the knife along the groove in the staff, and dividing the prostate and cellular membrane, leaves the rectum uncut. This valve of the rectum extends about an inch lower than the opening into the bladder." Dr. Muter observes, that it must be exceedingly difficult to avoid including the rectum also in this incision, in which case there would be no valve. And even granting that the surgeon succeeds in forming the valve, Dr. M. thinks that it would be in the way of extracting a large stone, forming a stricture of the wound, and impeding the exit of the calculus. Besides this, it would, he thinks, he very liable to be torn. The above objections of Dr. Muter, let it be remembered, are not from actual experience of their validity, but from reasoning on the subject.

16. *Mental Imbecility from injury of a Nerve.*—A young man, fourteen years of age, of plethoric habit and nervous temperament, received an injury on the top of the foot, from a stone thrown with violence by one of his playmates. By this accident a portion of the anterior tibial nerve, situated between the metatarsal bone of the great toe and the adjoining one, was contused. Only a slight degree of pain at first occurred, and that upon motion. About eight or ten weeks after the accident, the pain increased, accompanied by a slight swelling of the part, and also an extension of the pain to the ancle and leg. Lotions, fomentations, and rest, did no good, and the disease increased in violence, extending up along the trunk of the nerve, and affecting the adjacent muscles with spasms. The constitution now became involved in the irritation; the pulse increased in frequency and force, but the digestive organs continued in good order. The pain extended above the knee, and was almost insupportable. Large doses of assafœtida, conium, and even belladonna, produced no relief. The symptoms became more formidable; the spasmodic action of the extensor longus pollicis pedis, drew the great toe perpendicular to the surface of the foot; and the slightest motion or touch would excite excruciating pain, lasting from three to five minutes. At the end of three months from the accident, the whole nervous system became involved in these spasmodic irritations, and the intellect began to suffer. The unfortunate patient lost the power of reasoning and recollection; was unable to distinguish visitors, or recognize his parents; in fact, his mind became idiotic. The pain, however, now extended up along and beyond the thigh, affecting spasmodically the muscles of respiration. Though his distress was most acute, yet he gave no utterance to his feelings. "While the paroxysms were on him, he would roll his fist, and imitate the actions of a pugilist, but with much greater violence and rapidity, often striking his nearest and best friends, and all around him." It was now determined to make a division of the anterior tibial nerve, as a last resource. It was accordingly divided above the ankle, from a belief that it would more effectually cut off the connection between the injured part and the nervous system at large. An incision was made about two inches in length, just on the outside of the tibia, and a separation made between the extensor longus pollicis pedis and the tibialis anticus, down to the artery and nerve. The latter was divided by a bistoury, and about an inch of it cut out. The extensor muscle of the great toe became immediately so relaxed, as to allow the toe to resume its natural situation by the side of the other toes. There was also an almost immediate alleviation of all those extraordinary morbid phenomena that had exhibited themselves in the unfortunate sufferer. The local affections became less distressing, and in a short time wholly disappeared. He has since enjoyed good health.

Although Dr. Anderson does not mention anything about the return of sound intellect, we take it for granted that this was the case, otherwise he would not have stated the enjoyment of *good health.* The case affords a curious instance of the power of a local affection in deranging the health of body and mind.—*N. Y. Med. and Phys. Journ.*

17. *Resuscitation.*—Our readers are aware that some discrepancy of opinion prevails respecting the *power* which we possess of restoring suspended animation. If we are to believe the accounts transmitted to the Humane Society, there would seem to be scarcely any limits to this power, in respect to the period which elapses between suspension and restoration of the vital phenomena. Mr. Brodie and some others in this country, on the other hand, are very sceptical respecting the said power of resuscitation, after the cessation of the heart's action, which they suppose (from experiments on animals) cannot continue for more than a few minutes after the respiratory function ceases. We confess that, from what we have actually seen of these resuscitations and attempts at resuscitation, (which have been not a few) we are led to the same views as are entertained by Dr. Paris and Mr. Brodie. Our contemporaries of the North are of a different opinion, and they have published an analysis of Dr. Roesler's thesis in support of their opinion. Of this analysis we shall present the prominent features, being desirous that the means of saving human life should neither be depreciated nor rated too highly.

The first inquiry instituted by Dr. Roesler, was to determine whether, in death by submersion, the air passages are really obstructed by mucus and water; a subject which has occasioned much controversy. The result of 45 experiments made by Dr. R. was, that in every instance a very small quantity of frothy mucus might be seen about the bifurcation of the trachea; but no water whatever; the quantity of mucus being wholly insufficient to produce the slightest obstacle to artificial inflation of the lungs. The attempt at washing out the mucus by means of warm water, was not merely useless, but injurious. Not so the following:

"This process was instituted, in order to determine the average number of recoveries by the inflation of temperate air, and the longest period of submersion after which animation might be restored; with the view of afterwards comparing with these *data* the corresponding results obtained with heated air. The plan pursued was the following: As soon as the animal was removed from the water, it was carefully dried, laid in a warm, but well-ventilated place, and rubbed, and finally swathed in flannel. The artificial inflation was then immediately begun, and was continued for half an hour or three quarters of an hours; after which, if no signs of resuscitation appeared, it was abandoned. At the same time the frictions were frequently resumed, the body was kept well warmed, and as soon as any sign of returning life appeared, stimulants were introduced into the stomach. The only important particular which has been omitted in his description, is the number of inflations; but as far as can be gathered from the details of the experiments, they appear to have been about twenty per minute."

The average number of recoveries was three to eight. And next comes the great question as to the period of submersion. We have already stated the opinion of Mr. Brodie and Dr. Paris. It may be, that the latter author has thrown too much of an air of ridicule over a grave subject, by comparing the stories of divers, and the reports from humane societies, with the ancient tales respecting "the nymphs and mermaids whose residence is in grottoes beneath the waves of the sea." But on the other hand, our northern contemporaries are forced to admit "that the reports which have emanated from humane societies, and various other quarters, are too sanguine in their expectations of success in cases of long-continued submersion." The first train of experiments were three in number—two on rabbits and one on a cat. The periods of submersion were 5 3-4, 9 1-2, and 11 3-4 minutes, and in all the three cases, resuscitation was effected. But, *audi alteram partem.* "He several times failed when the animals were taken out of the water instantly after they seemed to have expired." It appears to us, therefore, that as the above 3 experiments were the only ones our contemporaries could select out of forty-five, which were at all in support of their doctrines, they have very little indeed to boast of, granting that no fallacy crept in, or no coloring was given to the said experiments—fallacies and colorings which they themselves acknowledge to have often disfigured reports in this country, and which may (without prejudice be it spoken) occur in the reports of men in other climates also.

A part of the investigation relates to the relative value of cool and warm air in the treatment of suspended animation. The results appear favorable to the employment of heated air for the inflation of the lungs; "but enough has not been done to determine its precise value."

We have only one more observation to make on this subject, namely, that men are not cats or rabbits. Experiments on the inferior animals will rarely apply to the human species. We have seen rabbits digest a good belly-full of parsley with both par vagums cut, and streams of electricity passing constantly along the nerves. But how would such an experiment succeed in man?—*D. Roesler's Inaugural Dissertation.—Edinb. Journ.*

18. *Tympanitic state of the Pericardium.*—Mr. Scott of the Haymarket, aged about 47, had, for three or four years, been declining in health, but had not been under regular medical superintendence until a few weeks before his decease. No regular history of the complaint therefore could be obtained. He stated however, that his appetite and strength had gradually declined, but his chief complaint was, a fluttering, a palpitation, and a sense of anxiety about the region of the heart, with disturbed sleep, and frightful dreams. When seen a few weeks before death, his countenance was like that of a person in a state of anemia, except that there was also a chlorotic tinge in the skin. The pulse was very feeble, quick, and irregular; there was a tendency to œdema about the ancles; the appetite was almost entirely gone, and the patient felt approaches to syncope, on using any exertion, or ascending stairs. His mind was desponding, and temper irritable. The motions from his bowels were perfectly healthy. The chest in every part sounded remarkably well. In the region of the heart percussion elicited as clear a sound as in any other part of the chest. The impulse of the heart against the ribs was very feeble, and scarcely audible. It was also irregular, in correspondence with the state of the pulse. The patient was under the care of Mr. Fincham, of Spring Gardens, and was visited successively by Dr. Hooper, Dr. Macmichael, and Dr. Johnson.

When the above symptoms and phenomena were ascertained by the last-mentioned physician, (not in consultation with the others,) he gave it as his opinion to Mr. Fincham, that the patient labored under disease of the heart; and that the nature of the lesion was probably a weakening and degeneration of the muscular structure. In a few days after this examination, the patient suddenly died, and the body was examined by Dr. Johnson, Mr. Fincham, and Mr. Henry Johnson.

Dissection, 13th February, 1825.—The body extenuated, but still there was some peculiarly yellow fat on the chest and abdomen. The muscles, though wasted, were of a vivid red color. All the organs in the abdomen were sound. On opening the chest, the lungs presented a beautiful blue appearance, sparingly mottled with white; they were very sound. Between the two lungs there presented itself a pellucid membrane distended with air. This was found to be the pericardium, reduced to a most extraordinary degree of tenuity, and distended with a very considerable quantity of air. The heart was small (not half filling the pericardium) and extremely degenerated in substance, a great part of its muscular structure being converted into a kind of fat. The whole was so lacerable as scarcely to bear handling. The parietes of the left ventricle were not more than a quarter of an inch in thickness; the internal surfaces of the cavities were pale, wasted, and not containing a single drop of blood. Neither was any blood to be seen in the large vessels issuing from the heart. There was nothing particular in the valvular structure of the organ.

Dr. Johnson has met with several cases where the heart was in this degenerated condition, but never before observed such a distention of the pericardium by air. It is quite evident from the size of the pericardium, and its extreme tenuity, that this collection of air must have been of some standing. This phenomenon accounts for the region of the heart being as sonorous as any other part of the chest, which is not usually the case.—*Med. Chi. Rev.*

19. *Dropsy in Pregnancy.*—Dr. Felici, of Milan, relates the case of a woman, thirty-five years of age, who considered herself pregnant in December, 1817, soon after which a tumefaction appeared in the feet, and spread to the thighs, pudenda, loins, and abdomen, attended with much thirst. On the 2d of February she was seized with fever, and acute pain in the left hypogastric region, with numbness of the corresponding thigh. These symptoms were combated by antiphlogistics and digitalis, except the thirst, which continued insupportable. The œdema increased; the swelling of the abdomen became enormous; the desire of making water violent and painful. The pain in the leg and thigh had greatly increased; there was difficulty of breathing; palpitations; a dry cough; wasting of the upper extremities; frequent pulse; sense of suffocation upon the slightest motion; puffiness of the face. Such was the poor woman's state, when seen, for the first time, by Dr. Felici, on the 6th of March. The doctor suspecting retention of urine, introduced the catheter, and drew off 13 *or* 14 *pints of urine!* This operation, repeated twice in the same day, produced the evacuation of an equal quantity. Next day the symptoms were mitigated: the catheter was passed twice. The fever and thirst continued; but the pain in the side was gone; the thigh could be moved; and the œdema of the lower extremities greatly diminished. On the 8th a purgative was taken, which procured several watery evacuations; but no urine discharged. The catheter again drew off as large a quantity as on the 6th. About noon labour came on; the fœtus and placenta were thrown off without accident, and there was no subsequent lochia.

The catheter was used every two hours on the 7th, 10 h, 11th, and 12th. On the 13th the abdominal swelling was entirely gone, as was that of the right thigh. It was not till the 18th that the painter was able to make water naturally, for the first time, and by the 31st was able to lay aside the catheter.

This was one of those cases which we occasionally see of immense distention of the urinary bladder. There was a man last year in St. George's Hospital, who was supposed to labor under ascites, and paracentesis abdominis was in contemplation, when the house-surgeon happened to introduce a catheter, and drew off many quarts of water, with immediate reduction of the abdominal swelling.—*Ib.*

